# Four new endemic genera of Rubiaceae (Pavetteae) from Madagascar represent multiple radiations into drylands

**DOI:** 10.3897/phytokeys.99.23713

**Published:** 2018-05-21

**Authors:** Petra De Block, Franck Rakotonasolo, Salvator Ntore, Sylvain G. Razafimandimbison, Steven Janssens

**Affiliations:** 1 Botanic Garden Meise, Nieuwelaan 38, 1860 Meise, Belgium; 2 Kew Madagascar Conservation Centre, Lot II J 131 Ambodivoanjo, Ivandry, Antananarivo, Madagascar; 3 Parc Botanique et Zoologique de Tsimbazaza, Antananarivo-101, Madagascar; 4 Swedish Museum of Natural History, Department of Botany, P.O. Box 50007, SE-10405 Stockholm, Sweden

**Keywords:** *Coptosperma*, dry forests, endemism, fruits, generic delimitation, Madagascar, Pavetteae, placentation, pollen, pyrene opening mechanisms, radiation, rumination, seeds

## Abstract

The taxonomic positions and phylogenetic relationships of six Pavetteae species endemic to Madagascar were tested with a phylogenetic study of the Afro-Madagascan representatives of the tribe Pavetteae based on sequence data from six markers *rps16*, *trnT-F*, *petD*, *accD*-*psa1*, *PI* and ITS. The six species were resolved into four well-supported and morphologically distinct clades which we here formally recognise at generic level. The new genera are the monospecific *Exallosperma* and *Pseudocoptosperma*, each with a single species, and *Helictosperma* and *Tulearia*, each with two species. Each genus is characterised by one or more autapomorphies or by a unique combination of plesiomorphic characters. Mostly, the distinguishing characters are found in fruit and seed; *Exallosperma* differs from all other Pavetteae genera by the fruit consisting of two stony pyrenes, each with a single laterally flattened seed with irregularly distributed ridges on the surface; *Helictosperma* is unique by its single spherical seed rolled-in on itself in the shape of a giant pill-millipede. *Pseudocoptosperma* is characterised by the combination of three ovules pendulous from a small placenta and triangular stipules with a strongly developed awn, whereas *Tulearia* is characterised by robust sericeous flowers, small leaves, uni- or pauciflorous inflorescences and fruits with two pyrenes, each with a single ruminate seed.

The four new genera show marked adaptations to the dry habitats in which they grow. They represent multiple radiations into drylands and highlight the importance of the dry forest and scrub vegetation in western, southern and northern Madagascar for plant biodiversity. The description of the four new genera shows that the tribe Pavetteae exhibits the same pattern as many plant groups in Madagascar, which are characterised by a high proportion of endemic genera comprising a single or a few species.

In the four new genera, five new species are described and one new combination is made: *Exallosperma longiflora* De Block; *Helictosperma malacophylla* (Drake) De Block, *Helictosperma poissoniana* De Block, *Pseudocoptosperma menabense* Capuron ex De Block; *Tulearia capsaintemariensis* De Block and *Tulearia splendida* De Block.

## Introduction

With ca. 750 species, the Pavetteae is one of the largest tribes of subfamily Ixoroideae. The tribe is paleotropical and comprises the species-rich genera *Pavetta* L. (ca. 400 species) and *Tarenna* Gaertn. (ca. 200 species). The tribe has three main centres of distribution, notably the Asian-Pacific region with ca. 280 species belonging to four genera, continental Africa with ca. 350 species belonging to eight genera and Madagascar. In Madagascar, the tribe is represented by ca. 80 species (De Block, pers. obs.) and six genera are hitherto described. The Pavetteae are characterised by interpetiolar stipules, absence of raphides, terminal inflorescences, secondary pollen presentation, corolla lobes contorted to the left, 3(-4)-colporate tectate pollen grains, fleshy fruits, seeds with an adaxial excavation and exotestal cells either parenchymatic or with thickenings mainly along the outer tangential wall (Bridson and Robbrecht 1985, Robbrecht 1988, De Block 1997; De Block and Robbrecht 1998).

With ca. 80 species, the Pavetteae account for ca. 10% of the Madagascan Rubiaceae species, estimated at ca. 800 species (Govaerts et al. continuously updated). They are widely distributed in both dry and humid forests and are small shrubs or trees with usually large inflorescences and, often sizeable, white flowers. The Madagascan Pavetteae show great diversity in fruit and seed structure, placentation and pollen type (Bridson and Robbrecht 1985, De Block 1997, De Block and Robbrecht 1998, De Block et al. 2001), characters that usually are more conservative at tribal level. Many species remain undescribed, some of which cannot be easily accommodated in the currently recognised genera of the tribe.

Recently, the first molecular phylogenetic study of the Pavetteae (De Block et al. 2015) confirmed the monophyly of the tribe and identified four main lineages, all strongly supported as monophyletic although their phylogenetic relationships remained unresolved. Two of these lineages were restricted to continental Africa. The first continental African clade comprises the genera *Nichallea* Bridson and *Rutidea* DC., while the second one contains the genus *Leptactina* Hook.f. including *Coleactina* N.Hallé and *Dictyandra* Welw. ex Hook.f. The third main lineage within the Pavetteae consists of the monospecific East African genus *Cladoceras* Bremek., the continental African species of the genus *Tarenna* and the paleotropical genus *Pavetta*. The fourth lineage includes the East-African monospecific genus *Tennantia* Verdc., the Asian-Pacific species of *Tarenna* and all Madagascan Pavetteae, some of which are also represented in continental Africa and the Indian Ocean islands (e.g. *Coptosperma* Hook.f., *Paracephaelis* Baill.). There was strong support for the monophyly of the genera *Robbrechtia* De Block *Homollea* Arènes and *Paracephaelis* sensu lato (including *Homolliella* Arènes). On the other hand, neither the largest Madagascar-centred genus *Coptosperma* nor the paleotropical genus *Tarenna* was recovered as monophyletic. In fact, the phylogeny of the Madagascan Pavetteae was largely unresolved due to low sequence divergence which is in contrast with the high morphological variation present in the group (De Block et al. 2015). This phenomenon is encountered in more (Madagascan) plant groups and is interpreted as the result of recent rapid radiation (e.g. Malcomber 2002, Mort et al. 2007, Janssen et al. 2008, Knope et al. 2012, Tosh et al. 2013). It should be noted, though, that recent studies using next generation sequencing (e.g. GBS) have managed to considerably boost resolution and support in hitherto badly resolved groups, such as *Coffea* L. (Hamon et al. 2017). In future, these new methods may also help resolve the phylogeny of the Pavetteae.

This study focuses on the fourth lineage of De Block et al. (2015), hereafter called the Afro-Madagascan clade. We add to the analysis six Madagascan endemics in order to assess their phylogenetic positions within the Afro-Madagascan clade. Five of these are species new to science, the sixth has been described in the genus *Ixora* L. and was later transferred to *Tarenna* [*Ixora malacophylla* Drake; *Tarenna malacophylla* (Drake) Homolle]. These species are clearly members of the Pavetteae because they have all the characters of the tribe as listed above. However, when comparing their characters with those of the currently recognised Madagascan genera of the Pavetteae (*Coptosperma*, *Homollea*, *Paracephaelis*, *Robbrechtia*, *Schizenterospermum* Homolle ex Arènes, *Tarenna*), they cannot be easily attributed to one of them. They have, for example, flower characters of one genus but fruit characters of another or they possess characters hitherto never encountered in the Pavetteae, which is especially the case for pyrenes and seeds.

This study aims to assess the taxonomic positions and phylogenetic relationships of these six Madagascan endemics through a combination of a molecular and a morphological study and to attribute to them a generic position. Can they be accommodated in existing Pavetteae genera or should new genera be described? The new species are described in detail and illustrations and distribution maps are given.

## Methods

### Taxon sampling

Two continental African species not belonging to the Afro-Madagascan clade of De Block et al. (2015) were chosen as outgroup taxa: *Tarenna precidantenna* (clade III of De Block et al. 2015) and *Leptactina mannii* (clade II of De Block et al. 2015). All sampled taxa of the Afro-Madagascan clade of De Block et al. (2015) were included for this study except for *Coptosperma* sp. nov. A. An additional species of the genus *Homollea* (*H. leandrii*) and 12 accessions of the six Madagascan species investigated were added, which brings the total number of our sampled Madagascan Pavetteae species to 30 out of a total of ca. 80. Except for *Helictosperma poissoniana*, at least two accessions for the six investigated species were included to test the species concept. Accession data of ingroup and outgroup taxa is given in Appendix 1.

### DNA isolation, amplification and sequencing

In addition to the markers *rps16*, *trnT-F* and ITS, which are the most used markers in Rubiaceae phylogenetic studies (Bremer 2009) and which were already used in De Block et al. (2015), we added sequencing data from *petD*, *accD-psa1* and *PI* (the floral homeotic gene PISTILLATA). The *petD* and *accD-psa1* markers are easy to amplify and have been used to resolve phylogenetic questions at lower taxonomic levels in Rubiaceae (e.g. for *accD-psa1*: Maurin et al. 2007; for *petD*: Janssens et al. 2016; for *accD-psa1* and *petD*: Tosh et al. 2009). *Pistillata* was not used before for phylogenetic analyses in Rubiaceae but has been proven successful in other groups such as, for example, Rosaceae (Oh 2013) and Brassicaceae (Bailey and Doyle 1999). *Pistillata* belongs to the B class genes of the family of MADS-box genes (low-copy nuclear genes) and is involved in flower development (Viaene et al. 2009). Other B class genes that have been successfully used for phylogenetic inference are the closely related *AP3*/*DEF* genes (Janssens et al. 2007, Volkmar et al. 2014, Geuten et al. 2006).

Total genomic DNA was extracted from silica-dried leaf material or herbarium material using either a modified version of the hot CTAB protocol (Saghai-Maroof et al. 1984, Doyle and Doyle 1987) or the commercial E.Z.N.A. High Performance Plant DNA Mini Kit (OMEGA bio-tek). Primers and PCR mixes for chloroplast *rps16*, *trnT-F* and nuclear ribosomal ITS are listed in De Block et al. (2015). Primers for *petD*, *accD*-*psa1* and *PI* are provided in Table 1. The PCR mixes for *petD* and *accD*-*psa1* contained 1 μl genomic DNA, 2 µl BSA, 2 µl MgCl_2_, 0.25 μl of each primer (100 ng/μl), 2 μl of 10 mM dNTPs, 2.5 μl dream Taq Buffer, 0.125 μl dream Taq DNA polymerase and were adjusted with MilliQ water to 25 μl. The PCR mix for *PI* is identical to that of *petD* and *accD*-*psa1* except for the use of 0.125 μl KapaTaq and 5 µl KapaTaq buffer. Amplification of *rps16*, *trnT-F* and ITS followed protocols of Oxelman et al. (1997), Razafimandimbison and Bremer (2002) and De Block et al. (2015), respectively. Amplification of *petD* was carried out using the following PCR programme: 95 °C for 3 min; 35 cycles of 95 °C for 30 s, 50 °C for 30 s, 72 °C for 2 min; and, a final extension of 72°C for 7 min. Amplification of *accD*-*psa1* was carried out following the same temperature profile but with an annealing temperature of 54 °C For *PI*, a touchdown PCR programme was used consisting of the following temperature profile: 95 °C for 3 min; 20 cycles of 95 °C for 30 s, primer annealing for 30 s at starting temperature 65 °C and lowering 0.5 °C/cycle, 72°C for 1 min; 15 cycles of 95 °C for 30 s, 55 °C for 30 s, 72 °C for 1 min; and, a final extension of 7 min at 72 °C Amplification reactions were carried out on a Perkin Elmer GeneAMP 9700 thermocycler or Eppendorf Mastercycler. Sequencing reactions were performed using the Big Dye Terminator 3.1 Cycle Sequencing kit (Applied Biosystems, Foster City, USA) on an Applied Biosystems 310 Genetic Analyzer or were sent to Macrogen Inc. (Seoul, Korea) for sequencing.

**Table 1.** Amplification primers used for *petD*, *accD-psa1* and *PI*.

Region	Primer	Primer sequence (5’-3’)	Reference
*petD*	petB1365F	TTGACYCGTTTTTATAGTTTAC	Löhne and Borsch (2004)
	petD738R	AATTTAGCYCTTAATACAGG	
*accD*-*psa1*	accD769F	GGAAGTTTGAGCTTTATGCAAATG	Tosh et al. (2009)
	PSA175R	AGAAGCCATTGCAATTGCCGGAAA	
*PI*	PAV_PI_EX1F	AACTCAAGCAACAGGCAGGT	De Block et al. (this study)
	PAV_PI_EX3Rb	CCTGAGCTCAATCTGCATGCTRTCA	

### Sequence alignment and phylogenetic analyses

It was impossible to obtain sequences for all accessions, especially for the markers *PI* and ITS. In case sequences could not be obtained, their positions in the dataset were regarded as missing data. *PI* sequences are missing for *Coptosperma madagascariense*, one of two *C. nigrescens* accessions, both *Exallosperma longiflora* accessions, one of two *Helictosperma malacophylla* accessions, *Homollea leandrii*, *Paracephaelis saxatilis*, *P. sericea*, *Tarenna attenuata*, *T. gracilipes*, *T. grevei*, *T. spiranthera* and one of three *Tulearia splendida* accessions. ITS sequences are missing for *Coptosperma* sp. nov. E, *Tarenna gracilipes* and two out of three *Tulearia splendida* accessions. Furthermore, ITS sequences for *Coptosperma madagascariense* and *Paracephaelis saxatilis* are from different accessions as the sequences of the other markers. Sequences of *rps16*, *TrnT-F* and *accD-psa1* are missing for *Homollea leandrii*. Lastly, *accD-psa1* and *petD* sequences are missing for *Tarenna attenuata* and for one out of three accessions of *Tulearia splendida*. Newly generated sequences have been submitted to GenBank (Appendix 1). Sequences were automatically aligned with MAFFT (Katoh et al. 2002) under the E-INS-I Algorithm with a scoring matrix of 100PAM/k=2 and a Gap open penalty of 1. The automatically aligned data matrix was subsequently finetuned by hand in the Geneious v. 5.6.1 software package.

The methodology of Wang et al. (2014) was used to infer topological conflicts between different datasets. A threshold with a BS value ≥ 70% and a PP value ≥ 0.95, was applied as an indication of strongly supported incongruence between different data sets. The best-fit nucleotide substitution model for each plastid and nuclear dataset was determined using jModelTest 2.1.4 (Posada 2008) under the Akaike information criterion (AIC). For ITS, *petD* and *PI*, the GTR+I+G model was found as best fit, whereas the GTR+G model was shown to be the best substitution model for *rps16* and *trnT-F* and the HKY+I model for *accD*-*psa1*. Bayesian analyses were conducted with MrBayes v. 3.1 (Huelsenbeck and Ronquist 2001) on three individual data partitions (plastid, *PI* and ITS) and a combined data matrix. Each analysis was run in parallel for 10 million generations with trees sampled every 1000 generations. Convergence of the chains was examined with TRACER v. 1.4 (Rambaut and Drummond 2007). Non-parametric bootstrapping was carried out with 100 bootstrap replicates.

### Taxonomy

Authors of species names are given in Appendix 1. Only for names not present there, authors are given in the text at the first use of the name. Herbarium material of the following institutions was studied: BR, G, K, MO, P, S, TAN, TEF, WAG and Z (Thiers continuously updated). Additional plant material and alcohol-preserved samples were collected during field work in Madagascar. Terminology follows Robbrecht (1988) but leaf shape is described according to the terminology of simple symmetrical plane shapes (Anonymous 1962). Methods follow normal practice of herbarium taxonomy (De Vogel 1987). Methods for pollen acetolysis follow De Block and Robbrecht (1998). In the descriptions, inflorescence size does not include the corolla tubes. For vegetative characters, colours and sizes are given for dried plant parts; for flower and fruit characters, colours are given for living material except when specified differently. Sizes of flowers and fruits cover the range of dried and alcohol-preserved material. Flowering and fruiting periods are based on dates given on the labels of herbarium material. Specimens are cited per province and alphabetically by collector. Material collected by staff of the Madagascan Service des Eaux et Forêts was given consecutive numbers in the series SEFM (suffix -SF) and CRNPNM (suffix -RN). When possible, the names of the individual collectors were retrieved from Dorr (1997). All specimens cited were seen, unless specified differently. Localities are cited as given by the collectors on the specimen labels. When no GPS coordinates were available, coordinates of localities were determined using the online Gazetteer to Malagasy Botanical Collecting Localities (Schatz et al. 2003). In addition, 1:500.000 maps from the Madagascan Institut National de Géodésie et Cartographie (Sarinantanin’i Madagasikara, 11 maps, fourth edition, 1990) were used to find geographical coordinates. Distribution maps were drawn using QGIS Desktop 2.14.20. Preliminary conservation status was assessed by applying the IUCN Red List Category criteria (IUCN 2017) using GeoCAT (Geospatial Conservation Assessment tool; Bachman et al. 2011). In Madagascar, many regions are undercollected. In order to compensate for an inadequate level of sampling, cell size has been chosen at 3×3 km, rather than the 2×2 km cell size suggested by IUCN for most taxa (Callmander et al. 2007).

Abbreviations used: col. ignot., collector unknown; fl., flowering; fr., fruiting; PK, point kilométrique; RN, Route Nationale; s.dat., without date; s.loc., without locality; st., sterile.

## Results

### Phylogenetic analysis

**Figure 1.**
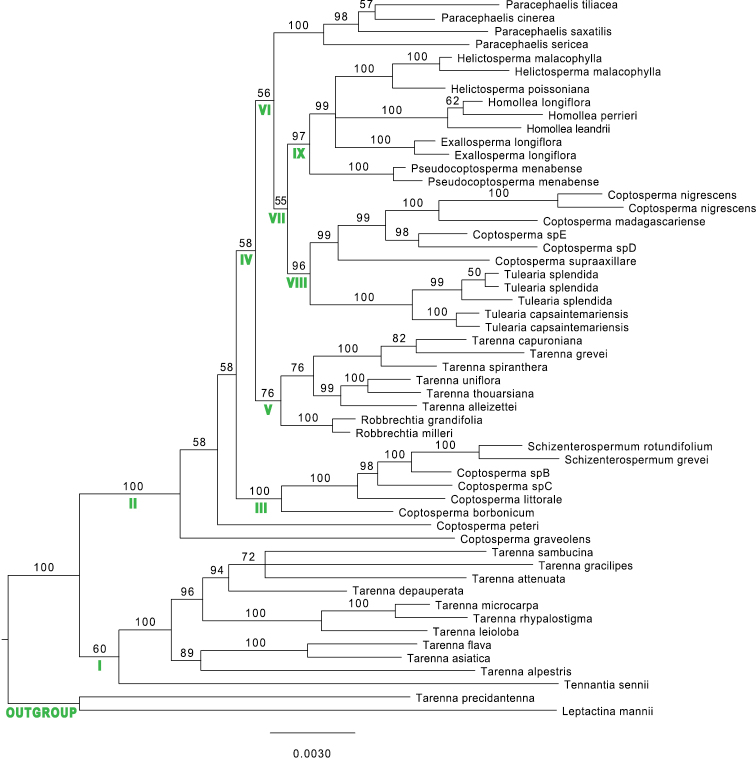
BI phylogram of the Afro-Madagascan Pavetteae clade and two outgroup taxa using *rps16*, *trnT-F*, *petD*, *accD*-*psa1*, *PI* and ITS sequences. BPP support is indicated.

For this study, we generated 176 new sequences, which were complemented with 121 sequences from GenBank, representing a total of 54 accessions and 48 species (see Appendix 1). Sequence variation within the individual datasets is summarised in Table 2. The majority-rule consensus topologies from the separate BI analyses of the *rps16*, *trnT-F*, *petD*, *accD*-*psa1*, *PI* and ITS data revealed similar topologies, yet did not provide a solid resolution for the majority of the clades. No hard incongruences were observed between the different datasets and they were combined for further analyses. The combined phylogeny of the six markers is shown in Fig. 1.

**Table 2.** Characteristics of individual datasets.

	*rps16*	*trnT-F*	*petD*	*accD-psa1*	ITS	*PI*
Number of sequences	53	53	52	51	50	41
Number of characters	888	1981	1054	1189	873	612
Constant characters	828	1811	969	1084	699	445
Variable characters	60	170	85	105	174	167

The monophyly of the ingroup, which corresponds to the Afro-Madagascan clade of De Block et al. (2015), is strongly supported (BPP = 100). Within the ingroup, two main clades are present, clades I and II (Fig. 1). Clade I is poorly supported (BPP = 60) and comprises the continental African monospecific genus *Tennantia* as sister to a clade formed by the Asian-Pacific species of *Tarenna* (BPP = 100).

Clade II (BPP = 100) comprises all Madagascan Pavetteae together with a few species from continental Africa and the Indian Ocean Islands. While the basal nodes in this clade are poorly supported (BPP < 60), there is strong support for more distal nodes. Within clade II, the East-African *Coptosperma graveolens* is sister to a clade comprising the rest of the taxa; within that latter clade, the East-African *C. peteri* is poorly supported as sister to all other taxa (BPP = 58). Clade III is a strongly supported monophyletic clade (BPP = 100), comprising two Madagascan *Schizenterospermum* species, two Madagascan *Coptosperma* species and *C. littorale* and *C. borbonicum* from continental Africa and the Mascarenes, respectively. Clade III is sister to clade IV (BPP = 58), which comprises the rest of the taxa included in this study. Within clade IV, two subclades V and VI are weakly supported. In clade V (BPP = 76), *Robbrechtia* is strongly supported as monophyletic (BPP = 100) and sister to a clade comprising the Madagascan representatives of *Tarenna* (BPP = 76). The Madagascan *Tarenna* species are grouped in two well-supported clades, one comprising *T. capuroniana*, *T. grevei* and *T. spiranthera* (BPP = 100) and the other comprising *T. uniflora*, *T. thouarsiana* and *T. alleizettei* (BPP = 99). Clade VI (BPP = 56) is subdivided into *Paracephaelis*, which is strongly supported as monophyletic (BPP = 100), and a weakly supported clade VII (BPP = 55), which comprises all newly included species studied here. Clade VII consists of two strongly supported subclades VIII (BPP = 96) and IX (BPP = 97).

Clade VIII comprises a well-supported subclade (BPP = 99) of *Coptosperma* species, consisting of the type species *C. nigrescens* as well as *C. madagascariense*, *C. supra-axillare* and two undescribed Madagascan species. *Coptosperma nigrescens* and *C. supra-axillare* occur in Madagascar, on the African mainland and in the Comoros (both species) and the Seychelles (*C. supra-axillare*), whereas the other species in this subclade are endemic to Madagascar. Sister to this *Coptosperma* clade is the *Tulearia* clade, which is strongly supported as monophyletic (BPP = 100) and comprises two undescribed species endemic to Madagascar. Within clade IX, the *Pseudocoptosperma* clade, comprising a single Madagascan species new to science, is sister to a polytomy of three subclades (BPP = 99). While the relationships amongst these subclades remain unclear, all three are strongly supported as monophyletic (BPP = 100). These three subclades comprise the genus *Homollea* and the *Exallosperma* and *Helictosperma* subclades. These latter two are made up of, respectively, one and two species endemic to Madagascar.

### Taxonomy

Four new genera with five new species are described here. One new combination is made.

#### Exallosperma

Taxon classificationPlantaeGentianalesRubiaceae

De Block gen. nov.

urn:lsid:ipni.org:names:77178881-1

##### Diagnosis.

Unique within the tribe Pavetteae by the pollen with psilate tectum and by the fruit containing 2 stony pyrenes, each with a laterally flattened ovoid seed with irregularly distributed surface ridges formed by elongation of the exotesta cells.

##### Type species.

*Exallosperma longiflora* De Block.

Shrubs, with *Terminalia*-branching pattern, branching modules consisting of a long-shoot, horizontal in orientation, never bearing inflorescences and relatively smooth, and an inflorescence-bearing short-shoot with short internodes, erect in orientation, densely beset with corky stipular remnants and alternating vegetative and reproductive nodes; vegetative parts pubescent. Leaves grouped terminally on short-shoots, deciduous, petiolate with petioles long, slender and canaliculate above; blades papyraceous; hair tuft domatia present; margins not revolute; bases rounded, subcordate, cordate or unequal, more rarely truncate or obtuse. Stipules keeled, with a dense row of large colleters interspaced with hairs at the base but otherwise glabrous on the inner surface except for the tip, dimorphic: in vegetative nodes consisting of truncate or triangular sheaths forming a cone and topped by needle-like awns, in inflorescence-bearing nodes consisting of ovate sheaths with acute or shortly acuminate tips. Inflorescences seemingly terminal but actually pseudo-axillary on erect short-shoots, pedunculate, pauciflorous, cymose with trichotomous branching; all parts (axes, bracts, bracteoles, pedicels) pubescent; bracts and bracteoles well-developed, linear. Flowers hermaphroditic, pentamerous, shortly pedicellate; all parts (ovary, calyx, corolla) pubescent outside; secondary pollen presentation present. Calyx well-developed; tube short; lobes much longer than tube. Corolla white, turning yellowish with age; tube narrowly cylindrical; lobes contorted to the left in bud and spreading at anthesis. Stamens sessile, inserted in the sinuses of the corolla lobes somewhat below the level of the throat; anthers almost completely included in the corolla tube at anthesis, basimedifixed, with sagittate base and short sterile apical appendix. Disc annular, fleshy, glabrous. Ovary cup-shaped, bilocular; placentation axile, with 3–4 ovules arising on top of a small placenta attached to the base of the septum. Style and stigma only just exserted from the corolla tube at anthesis; stigmatic lobes slender, fused over their entire length except for the very tips, receptive zone on the adaxial surfaces of the free tips and along the lines of fusion of the lobes. Fruits drupaceous, ovoid, pubescent, crowned by the persistent calyx, containing 2 pyrenes; pyrene stony, hemi-ellipsoid with the abaxial side convex and the adaxial side consisting of a flat rim but otherwise open (with the openings of the two pyrenes inside a fruit separated by the membraneous septum), with a short apical longitudinal preformed germination slit on both abaxial and adaxial sides, containing 1 seed; seed laterally flattened, ± bean-shaped; hilum superficial, irregularly ovate, moderate annulus around hilum present; seed surface not smooth but with irregularly distributed ridges formed by the seed-coat; exotesta cells with continuous plate-like thickenings along the outer tangential and upper parts of the radial walls, irregular ridges on seed surface formed by strongly elongated exotesta cells; endotesta consisting of crushed cell layers with many crystals; endosperm entire. Pollen grains 3-zonocolporate, exine psilate, supratectal elements absent.

A monospecific genus, endemic to northern Madagascar, occurring on calcareous soil.

##### Etymology.

This genus is named for its peculiar seeds.

#### Exallosperma longiflora

Taxon classificationPlantaeGentianalesRubiaceae

De Block sp. nov.

urn:lsid:ipni.org:names:77178882-1

**Figure 2.**
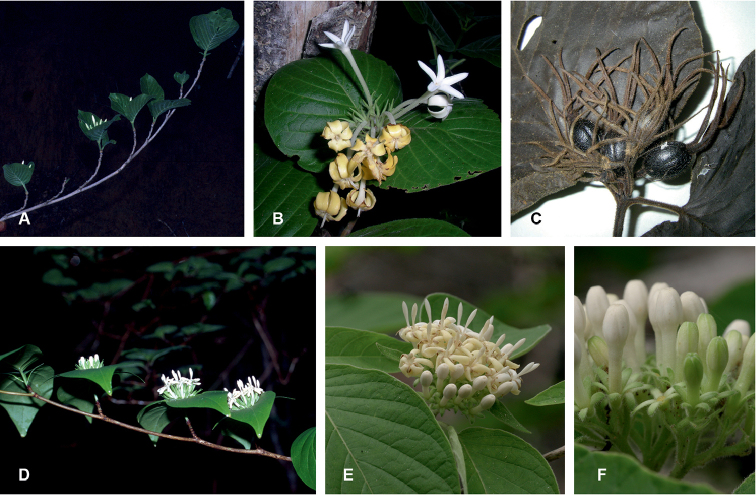
*Exallosperma* and *Helictosperma*. **A–C** *Exallosperma longiflora*: **A** flowering branch **B** inflorescence **C** infructescence from herbarium specimen Gautier et al. 4257 **D** *Helictosperma poissoniana*, flowering branch **E, F** *Helictosperma malacophylla*: **E** inflorescence **F** detail of inflorescence. Photographs: P. De Block (**A, D**), S. Dessein (**E, F**), L. Nusbaumer (**B, C**, ©: Conservatoire et Jardin botaniques de la Ville de Genève).

Figs 2A–C , 4 , 5 , 9A–D, M

##### Diagnosis.

Differing from *Homollea septentrionalis* De Block by the size and shape of the leaves of the first order bracts (broadly ovate to orbiculate, 5.5–9.5 × 4.2–9.5 cm vs. broadly ovate to ovate, 0.8–3.5 × 0.5–2.5 cm in *H. septentrionalis*), the calyx tube and lobes which are glabrous inside (vs. densely sericeous), the lower number of ovules (3–4 vs. 4–6) and the different seeds (2 seeds with irregularly distributed surface ridges, ca. 8 × 5.5 mm vs. 2–6 seeds with smooth surface, ca. 4.5 × 2.5–3 mm).

##### Type.

MADAGASCAR. Antsiranana Province, Analamerana, bank of Irodo river, close to Irodo camp, 8 Jan. 2002 (fl.), De Block, Rakotonasolo & Randriamboavonjy 1132 (holotype: BR!; isotypes: BR!, G!, K!, MO!, P!, TAN!, UPS!).

Shrub, up to 5 m tall. Young shoots bisulcate, brown, densely covered with erect hairs, rapidly becoming corky with loss of pubescence; older branches brown or greyish-brown, corky and somewhat flaking. Leaves often immature at time of flowering, 7–12 × 5.5–8.5 cm, ovate or elliptic, more rarely broadly elliptic or broadly ovate (but leaves of first order bracts broadly ovate to orbiculate); blades papyraceous, drying brown to dark brown, not discolorous, densely covered with erect hairs on both surfaces; base cordate, rounded, truncate or unequal; apex acuminate, acumen 2–15 mm long; midrib and secondary nerves raised on the lower leaf surface; midrib impressed especially in the basal half on the upper leaf surface; 8–12 secondary nerves on each side of the midrib. Petioles densely covered with erect hairs, 10–25 mm long (but shorter in leaves of first order bracts). Stipules caducous, covered with erect hairs along the base and the keel outside but rapidly becoming corky and losing the pubescence; stipules of vegetative nodes with sheaths 1.5–2.5 mm long and awns 1.5–3 mm long, those of inflorescence-bearing nodes ovate with acute or shortly acuminate tips, 4–8 mm long. Inflorescences consisting of 3–12 flowers, 1–2 × 1–2 cm; anthesis asynchronous within inflorescence; all inflorescence parts (peduncle, axes, pedicels, bracts and bracteoles) densely covered with erect hairs; peduncle 1–3 cm long; first order axes 3–10 mm long; first order bracts with stipular parts triangular and leaves broadly ovate to orbiculate, 5.5–9.5 × 4.2–9.5 cm, with strongly cordate or cordate bases and petioles 3–6(–10) mm long; higher order bracts linear, up to 1.6 cm long; bracteoles opposite on the pedicel just below the ovary, linear, 0.4–1 cm long. Flowers sessile or shortly pedicellate, pedicels 0–2 mm long. Calyx green, densely covered with erect hairs outside; tube ca. 1 mm long, glabrous and without colleters inside; lobes narrowly triangular, 12–16 × 1–1.5 mm (but shorter in young buds), densely covered with appressed hairs at the base and spreading or erect hairs in the upper half inside, bases not overlapping but closely joining, tips acute. Corolla tube 2.7–3.6 cm long, ca. 1.5 mm in diameter at the base, c. 3.5 mm in diameter at the throat, densely covered with erect hairs outside, upper half densely covered with erect hairs inside with pubescence continuing in the throat and on the base of the corolla lobes; lobes elliptic, 9–11 × ca. 3.5 mm, sparsely to moderately covered with erect hairs outside, densely covered with erect hairs at the base inside, margins densely ciliate, tips acute to apiculate. Anthers sessile, inserted in the sinuses of the corolla lobes 2–2.5 mm below the level of the throat, included in the corolla tube except for the very tips, 3–3.5 mm long. Ovary ca. 1.5 mm long, green, densely covered with erect hairs. Style and stigma white, exserted from the corolla tube for 2–5 mm at anthesis, style glabrous or with a few long spreading hairs in the upper half; stigma slender, papillae present on the inner surface of the free tips, longitudinal papillate lines running down for up to 16 mm, but papillae absent just below the tips. Fruits 7–10 × 5–8 mm (persistent calyx not included), moderately to densely covered with erect hairs, drying black and glossy when ripe; seeds ca. 8 × 5.5 × 3 mm, dark brown.

##### Habitat.

Lowland dry deciduous and semi-deciduous forest on limestone; alt. 0–450 m.

##### Distribution.

*Exallosperma longiflora* is only known from the northernmost tip of Madagascar in the Sava and Diana Regions. Fig. 14A.

##### Phenology.

Flowering: January–February; Fruiting: April.

##### Critical note.

*Exallosperma* resembles the Madagascan endemic *Homollea* by the pedunculate, pauciflorous, pseudo-axillary inflorescences and the pentamerous flowers with relatively long corolla tubes and long, narrow calyx lobes. *Exallosperma* is characterised by the *Terminalia*-branching pattern, the large, broadly ovate to orbiculate leaves of the first order bracts, the basally attached placentas from which 3–4 collateral ovules arise, the fruit containing 2 stony pyrenes, each with a laterally flattened ovoid seed with irregularly distributed surface ridges formed by elongation of the exotesta cells and by the pollen with psilate tectum. *Exallosperma longiflora* may be confused with *Homollea septentrionalis*, which it resembles by the dense pubescence on vegetative and reproductive organs, the pauciflorous inflorescences, the long flowers with tapering corolla lobes and the long, linear calyx lobes. The two species can be distinguished by the size and shape of the leaves of the first order bracts (broadly ovate to orbiculate, 5.5–9.5 × 4.2–9.5 cm in *Exallosperma longiflora* vs. broadly ovate to ovate, 0.8–3.5 × 0.5–2.5 cm in *H. septentrionalis*), the pubescence of the calyx tube and lobes inside (glabrous vs. densely sericeous), the number of ovules (3–4 vs. 4–6) and the different seeds (2 seeds with irregularly distributed surface ridges, ca. 8 × 5.5 mm vs. 2–6 seeds with smooth surface, ca. 4.5 × 2.5–3 mm).

##### Preliminary IUCN assessment.

Endangered: EN B1ab(i, ii, iii, iv) + 2ab(i, ii, iii, iv). The extent of occurrence (EOO) of *Exallosperma longiflora* is estimated to be 1,791 km^2^ and its area of occupancy (AOO) 54 km^2^, which both comply with the criteria for the Endangered category under sub-criteria B1 and B2. The species is known from seven collections, all but two of these collected after the year 2000, reflecting the intensified collection effort in northern Madagascar during the last 20 years. *Exallosperma longiflora* occurs in four locations, three of which are within protected areas, notably Réserve Spéciale d’Andrafiamena (which includes Analamerana), Loky Manambato (Daraina) and Montagne de Français. The main threat to *E. longiflora* is decline of its habitat both inside and outside the protected areas as a result of slash-and-burn agriculture, logging for timber and charcoal and burning to favour the growth of young grass for the grazing of cattle. Furthermore, traditional mining for gold is a serious threath in the area (Rakotondravony 2009; Nusbaumer et al. 2010). Based on the above information, the species is listed as Endangered.

##### Additional specimens examined.

**MADAGASCAR**. **Antsiranana Province**: Montagne des Français, plateau supérieur de l’Anosiarivo, 28 Jan 1966 (fl.), Capuron 24425-SF (BR, P, TEF); Massif de l’Ankitakona, 25 Apr 1966 (fr.), Capuron 24663-SF (BR, P, TEF); Analamerana, bank of Irodo river, close to Irodo camp, 6 Jan 2002 (fl.), De Block, Rakotonasolo & Randriamboavonjy 1080 (BR, MO, P, TAN, UPS); Sava, sous-préfecture de Vohemar, commune rurale de Daraina, Daraina, forêt d’Ambilondomba, W of Ambilondomba, 300 m S du point côté 341, 150 m, 8 Mar 2003 (fr.), Gautier, Wohlhauser & Nusbaumer 4257 (BR, G, K); Sava, sous-préfecture de Vohemar, commune rurale de Daraina, Daraina, forêt de Solaniampilana-Maroadabo, à 700 m du point côté 608, au 85°, 437 m, 2 Feb 2006 (fl.), Nusbaumer & Ranirison 1992 (BR, G); Sava, sous-préfecture de Vohemar, commune rurale de Daraina, Daraina, forêt de Solaniampilana-Maroadabo, à 750 m du point côté 608, au 205°, 328 m, 4 Feb 2006 (fl.), Nusbaumer & Ranirison 2151 (G).

#### Helictosperma

Taxon classificationPlantaeGentianalesRubiaceae

urn:lsid:ipni.org:names:77178883-1

##### Diagnosis.

Differing from *Exallosperma* by the shorter calyx lobes (3–9 mm vs. 12–16 mm long), the shorter corolla tubes (0.7–1.4 cm vs. 2.7–3.6 cm long), the completely exserted anthers at anthesis (vs. included in the corolla tube except for the tips), the pollen with microreticulate to perforate tectum (vs. psilate tectum), the fruits containing a single stony pyrene that opens into four valves, and the single seed that is rolled-in on itself like a giant pill-millipede (vs. fruits containing 2 hemi-ovoid pyrenes not opening into 4 valves, each with 1 laterally flattened, bean-shaped seed).

##### Type species.

*Helictosperma malacophylla* (Drake) De Block

Shrubs or small trees, with *Terminalia*-branching pattern, branching modules consisting of a long-shoot, horizontal in orientation, never bearing inflorescences and relatively smooth, and an inflorescence-bearing short-shoot with short internodes, erect in orientation, densely beset with corky stipular remnants and alternating vegetative and reproductive nodes; vegetative parts glabrous or pubescent. Leaves grouped terminally on short-shoots, deciduous, petiolate with petioles long, slender and canaliculate above; blades papyraceous; domatia present; margins not revolute; bases rounded, subcordate, cordate or unequal, more rarely truncate or obtuse. Stipules keeled, with a dense row of large colleters interspaced with hairs at the base but otherwise glabrous on the inner surface, dimorphic: in vegetative nodes consisting of truncate or triangular sheaths forming a cone and topped by needle-like awns, in inflorescence-bearing nodes consisting of ovate sheaths with acute or shortly acuminate tips. Inflorescences seemingly terminal but actually pseudo-axillary on erect short-shoots, pedunculate, pauci- or multiflorous, cymose with trichotomous branching; all parts (axes, bracts, bracteoles, pedicels) glabrous or pubescent; bracts and bracteoles well-developed, linear. Flowers hermaphroditic, pentamerous, pedicellate; all parts (ovary, calyx, corolla) glabrous or pubescent outside; secondary pollen presentation present. Calyx well-developed; tube short; lobes much longer than tube. Corolla white, turning yellowish with age; tube narrowly cylindrical; lobes contorted to the left in bud and spreading at anthesis, oblong, with blunt and emarginate tips. Stamens inserted in the sinuses of the corolla lobes at the level of the throat; filaments short; anthers completely exserted from the corolla tube at anthesis, basifixed, with sagittate base and short sterile apical appendix. Disc annular, fleshy, glabrous. Ovary cup-shaped, bilocular; placentation axile, with 3 ovules arising on top of a small placenta attached to the lower half of the septum. Style and stigma exserted from the corolla tube at anthesis; stigmatic lobes fused over their entire length except for the very tips, receptive zone on the adaxial surfaces of the free tips and along the lines of fusion of the lobes. Fruits drupaceous, spherical, pubescent or glabrous, crowned by the persistent calyx, containing 1 pyrene; pyrene crustaceous, spherical, formed by the outer convex parts of the two locules (the septum remaining membraneous and pushed to the side by the developing seed), opening along 4 preformed longitudinal germination slits of which 2 run down the margins of the locules and 2 are perpendicular to those, containing 1 seed; seed spherical, rolled-in on itself in the shape of a giant pill-millipede; hilum ovate, profound, moderate annulus around hilum present; exotesta cells with continuous plate-like thickenings along the outer tangential and upper parts of the radial walls, annulus formed by strongly elongated exotesta cells; endotesta consisting of crushed cell layers with many crystals; endosperm entire. Pollen grains 3-zonocolporate, exine microreticulate to perforate, supratectal elements absent.

A genus with 2 species, endemic to western and northern Madagascar, occurring on calcareous soil.

##### Etymology.

The genus is named for the shape of the seeds, which are rolled-in on themselves in the shape of giant pill-millipedes.

##### Key to the species of *Helictosperma*

**Table:** 

1	Vegetative and reproductive parts densely covered with erect or spreading hairs; inflorescences consisting of 25–90 flowers, 2.5–8 × 2–7 cm; calyx lobes 3–5 × 1–1.5 mm; corolla tube pubescent outside; corolla lobes with ciliate margins	***H. malacophylla***
–	Vegetative and reproductive parts usually glabrous but, if pubescent, then hairs appressed; inflorescences consisting of (1–)5–15(–20) flowers, up to 3 × 2 cm; calyx lobes 7–9 × 1.5–2.5 mm; corolla tube glabrous outside; corolla lobes without ciliate margins	***H. poissoniana***

#### Helictosperma malacophylla

Taxon classificationPlantaeGentianalesRubiaceae

(Drake) De Block comb. nov.

urn:lsid:ipni.org:names:77178884-1

Figs 2E, F , 6 , 8 , 9E–H, N

Ixora malacophylla Drake, Bull. Mens. Soc. Linn. Paris 2: 1309 (1897) & Hist. Phys. Madagascar, Atlas 4: t. 422 (1897). Tarenna malacophylla (Drake) Homolle, Bull. Soc. Bot. France 85: 606, fig. 1.5 (1938); Capuron, Rév. Rub. Mad. Com.: 173 (1973). Type: MADAGASCAR. s.loc., s.dat. (fl.), Grevé 112 (lectotype: P!, designated here; isolectotypes: BM!, K!, P!).

##### Description.

Shrub 2–6 m tall, more rarely tree up to 12 m tall with trunk up to 6 m tall and dbh up to 10 cm; young shoots quadrangular, often bisulcate, brown, densely covered with erect to spreading hairs; older branches brown, pale brown, greyish or fawnish, glabrous, often flaking. Leaves often immature at time of flowering, 6–15 × 4–8.5 cm, ovate, more rarely broadly ovate, elliptic or obovate; blades papyraceous, drying brown to dark brown, more rarely greenish-brown above, brown and often somewhat paler below, densely covered with erect hairs on the lower surface, moderately to densely covered with erect or spreading hairs on the upper surface, pubescence denser on the midrib and secondary nerves on both surfaces; base rounded, subcordate, cordate or unequal, more rarely truncate or obtuse; apex acuminate, acumen 3–18 mm long; hair tuft domatia present; midrib and secondary nerves raised on the lower leaf surface; midrib impressed especially in the basal half on the upper leaf surface; 10–14 secondary nerves on each side of the midrib. Petioles densely covered with erect hairs, 14–45 mm long. Stipules caducous; densely covered with erect hairs outside but rapidly becoming corky and losing the pubescence; stipules of vegetative nodes with sheaths 3–5 mm long and awns 3–6 mm long, those of inflorescence-bearing nodes ovate with acute to shortly acuminate tips, 4–6 mm long. Inflorescences consisting of 25–90 flowers, 2.5–8 × 2–7 cm; peduncle, inflorescence axes and pedicels densely covered with erect hairs; peduncle 1–5.5 cm long; first order axes up to 2(–4) cm long; first order bracts with stipular parts narrowly triangular and leaves long-petiolate and identical in shape and size to the vegetative leaves or somewhat smaller; second order bracts of the central axis often similar to the first order bracts but leaves considerably smaller and narrower with acute to attenuate base, 1–6.5 × 0.3–3.2 cm; second order bracts of lateral axes reduced or absent; higher order bracts and bracteoles linear, moderately to densely covered with erect hairs on both surfaces, no colleters present inside; bracts up to 1.2 cm long; bracteoles subopposite on the pedicel, 0.2–0.4 cm long; first order branching often shifted above the first order bracts (up to 1 cm higher); bracts sometimes adnate to axis for up to 5 mm. Flowers pedicellate, pedicels 1–5 mm long. Calyx green, moderately to densely covered with erect hairs outside; tube ca. 0.5 mm long, with a sparse ring of appressed hairs at the base but without colleters inside; lobes erect in young bud, but rapidly becoming reflexed, oblong, 3–5 × 1–1.5 mm, the upper half sparsely covered with erect hairs inside, bases not overlapping but closely joining, tips obtuse. Corolla tube 7–8 mm long, ca. 1 mm in diameter at the base, ca. 1.5 mm in diameter at the throat, moderately to densely covered with erect hairs outside, the upper 2/3 moderately to densely covered with erect hairs inside; lobes 4–5 × 3–3.5 mm, glabrous on both surfaces, margins ciliate. Anthers 3.5–5 mm long; filaments 1–1.5 mm long. Ovary 1–1.25 mm long, green, densely covered with erect hairs. Style and stigma white, exserted from the corolla tube for 7–10 mm at anthesis; style densely covered with spreading, upwardly directed hairs in the upper half; stigma with upper 4–5 mm fusiform, longitudinal papillate lines running down for a further 3–4 mm. Fruits 4–6 mm in diameter (persistent calyx not included), moderately to densely covered with erect hairs, drying brown and glossy when ripe; seeds 3–5 mm in diameter, dark brown.

##### Habitat.

Lowland dry deciduous and semi-deciduous forest on calcareous soil, usually on sand; alt. 30–800 m.

##### Distribution.

*Helictosperma malacophylla* is known from the Boeny, Betsiboka and Sofia Regions (Mahajanga Province), from the Ihorombe (Fianarantsoa Province) and from the Atsimo-Andrefana and Menabe Regions (Toliara Province). Fig. 14B.

##### Phenology.

Flowering: November–February(–April); Fruiting: (November–)January–May.

##### Vernacular names.

Ampale (dialect Masikoro; coll. ignot. 21707-SF); nofotrakoho (coll. ignot. 19382-SF); talinala (dialect Masikoro; coll. ignot. 21708-SF); voloiravy (Randriamiera 8770-RN); zamanimbato (Rakotovao 3898-RN).

##### Uses.

Construction wood for houses and cattle enclosures (coll. ignot. 19146-SF, 19382-SF, 21707-SF, 21708-SF); fire wood (coll. ignot. 21707-SF, 21708-SF).

##### Critical note.

*Helictosperma* resembles *Exallosperma* by the *Terminalia*-branching pattern, the pedunculate, pseudo-axillary inflorescences and the basally attached placentas from which three collateral ovules arise. The genera differ by pollen (tectum microreticulate to perforate in *Helictosperma* vs. psilate in *Exallosperma*) and fruit/seed characters (fruit with two pyrenes, each with a single laterally flattened seed with irregularly distributed surface ridges vs. fruit with single pyrene falling apart into four valves and containing a single seed that is rolled-in on itself). *Helictosperma malacophylla* resembles *E. longiflora* by the general hairiness of the whole plant but differs from it by the larger number of flowers per inflorescence (25–90 in *H. malacophylla* vs. 3–12 in *E. longiflora*), the longer pedicels (1–5 mm vs. 0–2 mm long), the shorter bracteoles (2–4 mm vs. 4–10 mm long) and the shorter corolla tubes (7–8 mm long vs. 26–37 mm long) and calyx lobes (3–5 mm long vs. 12–16 mm long).

##### Preliminary IUCN assessment.

Near Threathened: NT. The extent of occurrence (EOO) of *Helictosperma malacophylla* is estimated to be 273.476 km^2^, which falls outside any threat category, but its area of occupancy (AOO) is 261 km^2^, which complies with the Endangered category under the sub-criterion B2. The species occurs in ten locations and is known from more than fifty collections, twelve of which were collected recently (after 1989). The distribution of these recent collections coincides with the distribution of the older specimens (from 1892 till 1975), indicating that the species remains present throughout its original distribution area. Only few specimens were collected from protected areas, notably Ankarafantsika National Park, Tsingy de Namoroka Strict Nature Reserve and Kirindy Mitea National Park. Despite its large extent of occurrence, *Helictosperma malacophylla* is threathened locally by reduction of its habitat through slash-and-burn agriculture, logging for timber and charcoal and burning to improve grazing. Based on the above observations, the species is assessed as Near Threathened.

##### Additional specimens examined.

**MADAGASCAR**. **Mahajanga Province**: 2 km N of Tsarahasina, 30 m, 10 May 2006 (fr.), Andriamahay & Rakotoarisoa 1359 (K); canton Bemanevika, Analafaly forest, 6 km E of Marotaolana, 384 m, 10 May 2005 (fr.), Birkinshaw, Andrianjafy & Raha-Jean 1525 (BR, MO, P, TAN); Réserve Naturelle VII, Ankarafantsika, 120–150 m, s.dat. (fl.), coll. ignot. 30-SF (P); vallée de Marivoraona, village le plus proche Ambodifiakarana, canton Betsandraka, district Tsaratanana, bord E du sentier d’Ambatobe à Ambodifiakarana, 30 Nov 1958 (fr.), coll. ignot. 19146-SF (P, TEF); forêt d’Anatialabe, village le plus proche Kamakama, canton Ankirihitra, district Ambatoboeni, 30 Nov 1958 (fr.), coll. ignot. 19382-SF (P, TEF); Soalala district, Réserve Naturelle Intégrale de Namoroka (Réserve Naturelle 8), c. 38.5 km S of Soalala, 120 m, 2 Feb 2000 (fr.), Davis, Rakotonasolo & Wilkin 2520 (BR, K, TAN); forêt de Marohogo, 22 m, 13 Feb 1999 (fr.), De Block & Rakotonasolo 799 (BR, C, G, K, MO, P, TAN, WAG); Réserve Naturelle VIII, Tsingy de Namoroka, canton Andranomavo, district Soalala, 24 Apr 1952 (fl.), Rakotovao 3898-RN (P, TAN); Réserve Naturelle VIII, Tsingy de Namoroka, Andranomavo, district Soalala, 29 Dec 1952 (fl.), Rakotovao 4918-RN (P, TAN); Ambatofolaka, Réserve Naturelle VIII, Tsingy de Namoroka, canton Andranomavo, district Soalala, 28 Mar 1954 (fr.), Rakotovao 6154-RN (BR, P, TEF); Ambatofolaka, Réserve Naturelle VIII, Tsingy de Namoroka, canton Andranomavo, district Soalala, 26 Jan 1954 (fr.), Rakotovao 6239-RN (BR, P, TEF); canton Andranomavo, district Soalala, 25 Feb 1957 (fr.), Randriamiera 8770-RN (BR, P, TEF); canton Andranomavo, district Soalala, 10 Nov 1958 (fl.), Randriamiera 9724-RN (BR, P, TEF); **Fianarantsoa Province**: de Ihosy 47–49 km ad SE per viam ad Ivohibe in nemorosis parvis residuis juxta pascua ignita, 650–700 m, 5 Nov 1967 (fl.), Bernardi 11197 (G, K, P); bassin de la Menarahaka, près du carrefour des routes d’Ihosy à Ivohibe et Iakora, 650 m, 10 Feb 1963 (fr.), Capuron 22618-SF (P, TEF); haut bassin de la Menarahaka, E d’Ihosy, 5 Nov 1967 (fr.), Capuron 27850-SF (BR, P, TEF); vallée de la Menarahaka, E d’Ihosy, 19 Dec 1968 (fl.), Capuron 28479-SF (P, TEF); 10 km NE d’Ihosy entre Ihosy et Ambararata, 22 Feb 1970 (fr.), Capuron 29068-SF (BR, P, TEF); road Antananarivo-Ihosy, a few km before reaching Ihosy, 4 Jan 1999 (fl.), De Block & Rakotonasolo 534 (BR, K, MO, TAN); haute vallée de la Menarahaka, E d’Ihosy, 700–800 m, 28 Jan–10 Apr 1955 (fr.), Humbert 29886 (BR, P); **Toliara Province**: Sakaraha, commune Mahaboboka, Marotsiraka, forêt d’Analaraty, 469 m, 24 Mar 2013 (fr.), Andriamihajarivo, Miandry & Rakotoarivony 1879 (BR, MO, P, TAN); c. 10 km N of Befandriana-Sud, 150 m, 28 Nov 1962 (fl.), Appert 108 (MO, Z); Morombe district, Tanandava-Tatalavalo, 70 m, 10 Mar 1963 (fr.), Appert 114 (MO, Z); Fotivolo, Ankazobe, Feb 1963 (fr.), Bosser 17287 (BR, P, TAN); environs de Berenty, 18 Feb 1970 (fr.), Bosser 19934 (BR, P); Betsipotika, E de Morondava, 18 Jan 1962 (fl.), Capuron 20872-SF (BR, P, TEF); N de Dabara, Mahabo, 1 Apr 1970 (fr.), Capuron 29141-SF (BR, P, TEF); forêt de Mavozobe, village le plus proche Mavozobe, canton Befandriana-Sud, sous-préfecture Morombe, 22 Feb 1964 (fr.), coll. ignot. 21707-SF (TEF); forêt de Mavozobe, village le plus proche Mavozobe, canton Befandriana-Sud, sous-préfecture Morombe, 22 Feb 1964 (fr.), coll. ignot. 21708-SF (P); Kirindi forest, N part - Conoco, 7–16 m, 19 Jan 2007 (fl.), De Block, Rakotonasolo, Groeninckx & Dessein 2194 (BR, G, MO, P, TAN); Morondava, 1892 (fl.), Grevé s.n. (K); Morondava, close to site of baobabs amoureux, 27 m, 22 Jan 2007 (fl.), Groeninckx, Rakotonasolo, De Block & Dessein 132 (BR, G, MO, P, TAN, WAG); bassin de la Malio, affluent de Mangoky, près d’Ambalabe, 400–450 m, Nov 1946 (fl.), Humbert 19447 (BR, P); bassin moyen du Fiherenana entre Lambomakandro et Sakaraha, 400 m, 10 Dec 1946 (fl.), Humbert 19681 (BR, P); N of Tulear, near Mangoky river, 50 m, 1 Jan 1989 (fl.), Phillipson 3068 (BR, K, MO, P, TAN, WAG); Horombe, Beroroha, Tsivoko, forêt humide de Makay dans la zone de Menapanda, 495 m, 9 Dec 2010 (fr.), Rakotovao & Andriantiana 5558 (BR, MO, P, TAN); district Ankazoabo, commune Ankazoabo, canton Morafeno, village le plus proche Ampanihimahasoa, route Sakaraha-Ankazoabo, 12 km SE d’Ankazoabo, 599 m, 11 Mar 2004 (fr.), Randrianaivo, Ratodimanana, Razafindraibe, Randrianarisoa, Edodoky & Tsimanoa 1058 (BR, G); Sakaraha, Mahaboboka, canton Marotsiraka Betsileo, S of Ambinanintelo village and S of the intersection of the two rivers Bevoalavo and Andranoheza, 417 m, 21 Feb 2011 (fr.), Randrianasolo, Andriamihajarivo, Razanatsima, Rakotoarivony, Randrianarivony, Fagnarena, Bruno & Redilike 1417 (BR, MO, P, TAN); forêt d’Anosilamy, canton Beronono, commune Beronono, 448 m, 13 Jan 2010 (fr.), Razakamalala, Rakotovao & Andriantiana 5161 (BR, MO, P, TAN); **Unplaced localities**: forêt de Moailake, Feb 1892 (fl.), Douilot s.n. (P); Nandrosia, May 1897 (fr.), Perrier de la Bâthie 234 (P); Boiny, not readable further, Jan 1902 (fl.), Perrier de la Bâthie 1011 (BR, P); **Without locality**: s.dat. (fr.), Baron 4612 (K, P); Central Madagascar, s.dat. (fr.), Baron 4673 (K); s.dat. (fr.), Baron 4679 (P); s.dat. (st.), Douilot s.n. (P); s.dat. (fr.), Homolle 1427 (P); s.dat. (fr.), Homolle 1473 (P); s.dat. (fr.), Homolle 1495 (P).

#### Helictosperma poissoniana

Taxon classificationPlantaeGentianalesRubiaceae

Homolle ex De Block sp. nov.

urn:lsid:ipni.org:names:77178885-1

Figs 2D , 7 , 9I–L

##### Diagnosis.

Differing from *Helictosperma malacophylla* by the pauciflorous inflorescences [(1–)5–15(–20) vs. 25–90 flowers], the larger calyx lobes (7–9 × 1.5–2.5 mm vs. 3–5 × 1–1.5 mm), the glabrous corolla tube, the corolla lobes without ciliate margins and the usually glabrous vegetative and reproductive parts, but, if pubescent, then hairs appressed (vs. erect or spreading in *H. malacophylla*).

##### Type.

MADAGASCAR. Antsiranana Province, Analamerana, along Ambatabe river, 41 m, 7 Jan 2002 (fl.), De Block, Rakotonasolo & Randriamboavonjy 1095 (holotype: BR!; isotypes: BR!, K!, MO!, P!, TAN!, UPS!).

Shrub 1.5–4 m tall, more rarely small tree to 4 m tall, dbh to 7 cm; young shoots somewhat quadrangular and bisulcate, dark brown, glabrous or sparsely to densely covered with appressed hairs; older branches brown, pale or greyish-brown. Leaves often immature at time of flowering, 3–10 × 2–6 cm, ovate, rarely elliptic or obovate; blades papyraceous, drying brown to dark brown, more rarely greenish, hardly discolorous, glabrous or with midrib and secondary nerves sparsely to densely covered with appressed hairs, more rarely also higher order nerves pubescent on the lower surface, glabrous or sparsely to moderately covered with appressed hairs on the upper surface; base rounded, subcordate, cordate or unequal, more rarely truncate or obtuse; apex acuminate, acumen 3–10(–15) mm long; hair tuft or ciliate pit domatia present, sometimes also in the axils of secondary nerves; midrib and secondary nerves raised on the lower leaf surface; midrib impressed in the basal half on the upper leaf surface; 5–8 secondary nerves on each side of the midrib. Petioles glabrous to densely covered with short appressed hairs, 5–35 mm long. Stipules caducous, glabrous or sparsely to densely covered with appressed hairs outside, but rapidly becoming corky and losing the pubescence; stipules of vegetative nodes with sheaths 1.5–2.5 mm long and awns 2–5 mm long, those of inflorescence-bearing nodes ovate with acute to shortly acuminate tips, 4–7 mm long. Inflorescences consisting of (1–)5–15(–20) flowers, up to 3 × 2 cm; peduncle, inflorescence axes and pedicels glabrous or moderately to densely covered with appressed hairs; peduncle 0.5–3.5 cm long; first order axes up to 1.2 cm long; first order bracts with stipular parts narrowly triangular and leaves long-petiolate and identical in shape and size to the vegetative leaves or somewhat smaller; second order bracts of the central axis often similar to the first order bracts but leaves considerably smaller and narrower with acute to attenuate base, more rarely identical in shape to vegetative leaves with cordate or rounded base, up to 3.5 × 2.5 cm; second order bracts of lateral axes, higher order bracts and bracteoles linear, glabrous, ciliate or sparsely to moderately covered with appressed or spreading hairs on both surfaces, no colleters present inside; bracts up to 2.2 cm long; bracteoles subopposite on the pedicel, 0.2–1.2 cm long; first order branching often shifted above the first order bracts (up to 1 cm higher); bracts often adnate to axis for up to 5 mm. Flowers pedicellate, pedicels 1–6 mm long. Calyx green; tube 0.75–1 mm long, glabrous or more rarely moderately to densely covered with appressed hairs outside, glabrous and without colleters inside; lobes erect, leaf-like, 7–9 × 1.5–2.5 mm, glabrous inside and outside but with margins ciliate or more rarely sparsely covered with appressed hairs outside (mostly in basal half or along veins), bases not overlapping but closely joining, tips acute to obtuse. Corolla tube 5–14 mm long, ca. 1 mm in diameter at the base, ca. 2 mm in diameter at the throat, glabrous outside, densely covered with erect hairs except at the base and at the throat inside; lobes 4–5 × 3–3.5 mm, glabrous on both surfaces, margins not ciliate. Anthers 3–4 mm long; filaments 1–1.5 mm long. Ovary 1–1.5 mm long, faintly ribbed longitudinally when dry, green, glabrous or more rarely moderately to densely covered with appressed hairs. Style and stigma white, exserted from the corolla tube for 4–7 mm at anthesis; style densely covered with spreading, upwardly directed hairs over the whole length except for a further 2–3 mm. Fruits 5–7 mm in diameter (persistent calyx not included), with faint longitudinal ribs, glabrous or more rarely moderately to densely covered with appressed hairs, drying blackish and glossy when ripe; seeds ca. 5 mm in diameter, dark brown.

##### Habitat.

Lowland dry deciduous and semi-deciduous forest on limestone; alt. 0–450 m.

##### Distribution.

*Helictosperma poissoniana* is known from the Diana Region (Antsiranana Province) and from the Boeny and Melaky Regions (Mahajanga Province). Fig. 14C.

##### Phenology.

Flowering: October–January, May; Fruiting: January–December.

##### Vernacular names.

Hazontaka (Rakotovao 4081-RN); maroampotatra (Rakotovao 3914-RN); pitsopitsoka (Randriamiera 6722-RN); refeko (Leandri 573); tsarepepana (dialect Antakarana; Humbert 19013); voanievitra (Rakotovao 6240-RN).

##### Critical notes.

The three flowering specimens from the Tsingy de Bemaraha (Leandri 573 & 578; Jongkind 3415) have longer flowers (corolla tube 13–14 mm long) than all other specimens of this species (corolla tube 5–9 mm long). – Some specimens in the P herbarium were annotated as *Tarenna poissoniana* Homolle (e.g. Poisson 21). Capuron (1973) discussed this species in his unpublished treatment of the Madagascan Rubiaceae under the same name.

##### Preliminary IUCN assessment.

Near Threathened: NT. The extent of occurrence (EOO) of *Helictosperma poissoniana* is estimated to be 70,048 km^2^, which exceeds the upper limits for any threat category but its area of occupancy (AOO) is 198 km^2^, which falls within the limits for the Endangered category under the sub-criterion B2. The species occurs in seven locations and in three protected areas: Namoroka Strict Nature Reserve, Bemaraha National Park and Ankarana Special Reserve. *Helictosperma poissoniana* is widespread but threathened locally as a result of the reduction of its habitat through slash-and-burn agriculture, illegal logging and fires to improve grazing. Furthermore, artisanal sapphire mining in Ankarana Special Reserve is a serious problem. Based on the above observations, the species is assessed as Near Threathened.

##### Additional specimens examined.

**MADAGASCAR**. **Antsiranana Province**: Massif de l’Ankarana, 5 Nov 1990 (fl.), Bardot-Vaucoulon 238 (P); Massif de l’Ankarana, 17 Nov 1990 (fl., fr.), Bardot-Vaucoulon 303 (K, P); plateau de l’Ankarana, W de Mahamasina (Antanatsimanaja), 23 Apr 1963 (fr.), Capuron 22670-SF (BR, P, TEF); près de Marotaolana, Anivorano Nord, 4 Nov 1966 (fr.), Capuron 24543-SF (BR, P, TEF); district Ambilobe, village Ambilomagodro, km 114, montagne d’Ambohibe, grès de l’Isalo, 300 m, 8 Feb 1960 (fr.), Cours & Humbert 5705 (P); Ankarana, close to Apondrabe river, 82 m, 26 May 1999 (fr.), De Block, Rapanarivo & Randriamboavonjy 1042 (BR, G, K, MO, P, TAN, WAG); Ankarana, following the dry river Apondrabe, close to Mahamasina, 82 m, 27 May 1999 (fr.), De Block, Rapanarivo & Randriamboavonjy 1057 (BR, K, MO, P, TAN); Analamerana, along Ambatabe River, 41 m, 7 Jan 2002 (fl.), De Block, Rakotonasolo & Randriamboavonjy 1092 (BR, MO, TAN, UPS); Ankarana, near Mahamasina, perte d’eau, 82 m, 15 Jan 2002 (fr.), De Block, Rakotonasolo & Randriamboavonjy 1242 (BR, G, K, MO, TAN, WAG); Ankarana Special Reserve, c. 5 km NW of park village near Besaboba river, 90 m, 25 Apr 1993 (fr.), Harder, Merello, Razafimandimbison & Razafindrabaeza 1704 (MO, P, TAN); Diego-Suarez, Jan 1945 (fl.), Homolle 305 (P); Ambodimagodro, plateau de l’Ankarana, Dec 1938–Jan 1939, 250 m (fl.), Humbert 19013 (P); plateau de l’Analamera, 50–400 m, Jan 1938 (fl.), Humbert 19184 (P); collines et plateaux calcaires de l’Ankarana du Nord, 30–350 m, 24 Jan–29 Feb 1960 (fr.), Humbert 32468 (BR, P); collines et plateaux calcaires de l’Ankarana du Nord, colline S du jardin botanique 8, 30–350 m, 24 Jan–29 Feb 1960 (fr.), Humbert 32626 (BR, P); collines et plateaux calcaires de l’Ankarana du Nord, 30–350 m, 24 Jan–29 Feb 1960 (fr.), Humbert 32832 (P); Ankarana du Nord, Mar 1962 (fr.), Keraudren 1687 (P); Ankarana Réserve Spéciale, close to camp des Anglais, 180 m, 18 Feb 1994 (fr.), Lewis, McDonagh, Andrianarisata, Randriamabolona, Andiratsiferama & Bled 1125 (BR, K, MO, P, WAG); Réserve Spéciale d’Ankarana, Ambondromifehy, 11 Jan 2008 (fr.), Rakotonasolo 1164 (K); **Mahajanga Province**: Beanka, partie sud, Sarodrano, relevé linéaire B30, 429 m, 5 Mar 2012 (fr.), Bolliger, Hanitrarivo & Rakotozafy 278 (BR, G); forêt de Marohogo, près du village de Marohogo, 7 Apr 1965 (fr.), Capuron 24091-SF (BR, P, TEF); Soalala District, Réserve Naturelle Intégrale VIII, Tsingy de Namoroka, c. 40 km S of Soalala, 130 m, 3 Feb 2000 (fr.), Davis, Rakotonasolo & Wilkin 2533 (BR, K, TAN); district Antsalova, Tsingy de Bemaraha, Réserve Naturelle IX, near Ambodiria, 150 m, 17 Mar 2004 (fr.), Davis & Rakotonasolo 3122 (BR, K); forêt de Marohogo, 22 m, 13 Feb 1999 (fr.), De Block & Rakotonasolo 797 (BR, C, G, K, MO, P, TAN, TEF, WAG); forêt de Marohogo, 22 m, 13 Feb 1999 (fr.), De Block & Rakotonasolo 798 (BR, C, G, K, MO, P, TAN, WAG); environs de Majunga, 2–15 m, 28–30 Dec 1924 (fl.), Humbert 4046 (BR, P); Tsingy de Bemaraha, N of Manambolo river, 50 m, 28 Nov 1996 (fl.), Jongkind, Andriantiana & Razanatsoa 3258 (BR, K, WAG); Tsingy de Bemaraha, N of Manambolo river, 50 m, 6 Dec 1996 (fl.), Jongkind, Andriantiana & Razanatsoa 3415 (BR, K, WAG); Réserve Naturelle IX, Bemaraha, Antsingy Nord, 22 Nov 1932 (fl.), Leandri 573 (P); calcaires de l’Antsingy, vers Ambodiriana, E d’Antsalova, 100–150 m, 9 Feb 1960 (fr.), Leandri & Saboureau 3072 (BR, P); Antsingy d’Antsalova, Tsingy de Bemaraha, Réserve Naturelle IX, Jan 1975 (fr.), Morat 4837 (P, TAN); Beanka, partie nord, bord de la rivière Bokarano, 187 m, 18 Dec 2011 (fr.), Nusbaumer, Bolliger, Hanitrarivo & Rakotozafy 3202 (BR, G); environs de Majunga, May 1908 (fl.), Perrier de la Bâthie 3266 (P); Namoroka, Andranomavo, Ambongo, Oct 1905 (fl.), Perrier de la Bâthie 3634 (BR, P); environs de Majunga, May 1908 (fr.), Perrier de la Bâthie 3766 (P); Kamakama, sur le plateau de l’Ankarana, Oct 1901 (fl.), Perrier de la Bâthie 3777 (P); Majunga, 22 Dec 1904 (fr.), Poisson 21 (P); Antsalova, Réserve Naturelle Intégrale IX, Tsingy de Bemaraha, Ambodiriana, 14 Mar 2004 (fr.), Rakotonasolo, Davis & Maurin 767 (BR, K, TAN); Réserve Naturelle VIII, Tsingy de Namoroka, canton Andranomavo, district Soalala, 30 Apr 1952 (fr.), Rakotovao 3914-RN (P); Réserve Naturelle VIII, Tsingy de Namoroka, canton Andranomavo, district Soalala, 10 Jun 1952 (fr.), Rakotovao 4081-RN (P); Réserve Naturelle VIII, Tsingy de Namoroka, canton Andranomavo, district Soalala, 20 Nov 1953 (fr.), Rakotovao 5672-RN (BR, P); Ambatafolaka, Réserve Naturelle VIII, Tsingy de Namoroka, canton Andranomavo, district Soalala, 4 Feb 1954 (fr.), Rakotovao 6240-RN (BR, P, TEF); Beanka, partie centrale, Andoloposa, 358 m, 26 Mar 2012 (fr.), Rakotozafy, Bolliger & Hanitrarivo 97 (BR, G); Boeny, canton Andranomavo, district Soalala, 13 Oct 1954 (fl.), Randriamiera 6722-RN (P, TEF); Boeny, canton Andranomavo, district Soalala, 18 Jan 1955 (fr.), Randriamiera 7070-RN (BR, P, TEF); Boeny, canton Andranomavo, district Soalala, 25 Feb 1957 (fr.), Randriamiera 8771-RN (BR, P, TEF); Boeny, canton Andranomavo, district Soalala, 15 Apr 1957 (fr.), Randriamiera 8795-RN (BR, P, TEF).

#### Pseudocoptosperma

Taxon classificationPlantaeGentianalesRubiaceae

De Block gen. nov.

urn:lsid:ipni.org:names:77178886-1

##### Diagnosis.

Differing from species within the *Coptosperma* assemblage by the combination of the following characters: 3 ovules pendulous from a small placenta attached to the upper half of the septum and keeled triangular stipules with well-developed awn (vs. stipules not keeled and without awn, of the “bec du canard” type).

##### Type species.

*Pseudocoptosperma menabense* Capuron ex De Block

Shrubs; vegetative parts except for young shoots glabrous. Leaves persistent, petiolate with petioles short and canaliculate above; blades coriaceous; domatia absent; margins revolute. Stipules triangular with well-developed awns, keeled, with 2 or 3 rows of colleters at the base but otherwise glabrous on the inner surface. Inflorescences terminal, sessile, multiflorous, cymose with trichotomous branching; partial inflorescences compact; all parts (axes, bracts, bracteoles, pedicels) densely pubescent; bracts and bracteoles small, triangular. Flowers hermaphroditic, pentamerous, sessile to shortly pedicellate; all parts (ovary, calyx, corolla) glabrous outside; secondary pollen presentation present. Calyx with short tube and small lobes. Corolla white, turning yellowish with age; tube narrowly cylindrical, short; lobes contorted to the left in bud and spreading at anthesis. Stamens inserted in the sinuses of the corolla lobes at the level of the throat; filaments short; anthers completely exserted from corolla tube at anthesis, basifixed, with sagittate base and short sterile apical appendix. Disc annular, fleshy, glabrous. Ovary cup-shaped, bilocular; placentation axile, with 3 ovules pendulous from the base and the lateral sides of a small placenta attached to the upper half of the septum. Style and stigma exserted from the corolla tube at anthesis; stigmatic lobes fused over their entire length, receptive zone along the lines of fusion of the lobes. Fruits drupaceous, spherical, glabrous, crowned by the persistent calyx, containing 1 pyrene; pyrene crustaceous, spherical, formed by the outer convex parts of one developed and one aborted locule (the septum remaining membraneous and pushed to the side by the developing seed), with a small central apical protuberance on the adaxial side, opening along the line of fusion of the locules, containing 1 seed; seed subspherical; hilum superficial, ovate, annulus around hilum absent; exotesta cells parenchymatic and filled with tannins; endotesta consisting of crushed cell layers without crystals; endosperm ruminate. Pollen grains 3-zonocolporate, exine microreticulate to perforate, supratectal elements absent.

A genus with a single species, endemic to western Madagascar.

##### Etymology.

The genus is named for its resemblance to *Coptosperma*.

#### Pseudocoptosperma menabense

Taxon classificationPlantaeGentianalesRubiaceae

Capuron ex De Block sp. nov.

urn:lsid:ipni.org:names:77178887-1

Figs 10 , 13 A–C, J

##### Diagnosis.

Differing from *Coptosperma mitochondrioides* Mouly & De Block by the triangular, keeled stipules with a robust awn (vs. stipules of the “bec du canard” type with rounded tip) and the smooth fruits (vs. fruits with ca. 10 longitudinal ribs).

##### Type.

MADAGASCAR. Mahajanga Province, forêt Tsimembo, dans la concession Barthe, 19 Dec 1953 (fl.), Martin 8252-SF (holotype: P!; isotypes: BR!, TEF!).

Shrub or small tree to 8 m tall, dbh to 10 cm; young shoots bisulcate, dark brown, densely covered with short erect hairs; older branches pale brown or fawn, glabrescent, in dried condition strongly contrasting with the blackish-brown stipules and dark brown petioles. Leaves 5–12 × 1–2.5 cm, narrowly elliptic or narrowly obovate; blades coriaceous, drying glossy and brown or more rarely greenish above, somewhat paler and dull below, glabrous on both surfaces; base cuneate to attenuate; apex acuminate, acumen 5–12 mm long; midrib raised and secondary and tertiary nerves somewhat raised on the lower leaf surface; midrib impressed on the upper leaf surface; 10–16 secondary nerves on each side of the midrib. Petioles 2–6 mm long, glabrous. Stipules drying blackish-brown, rapidly becoming corky, caducous, triangular with the robust awn as long as or longer than the basal sheath, glabrous outside, glabrous but with 2–3 basal rows of colleters inside; sheaths 1–2.5 mm long; awns 2–4 mm long. Inflorescences consisting of numerous flowers, 1–3.5 × 2–7 cm, sessile; inflorescence axes, pedicels, bracts and bracteoles densely covered with short erect hairs, green but drying dark brown; bracts with stipular parts reduced and foliar parts triangular and vaulted, 1–2 mm long, densely covered with appressed hairs and with a basal row of colleters inside, margins ciliate; central first order bracts often with stipular parts reduced and foliar parts leaf-like, 0.5–4 × (0.2–)0.4–0.9 cm, elliptic or narrowly elliptic, base attenuate or cuneate, petiole 1–2 mm long; bracteoles at the base of the ovary, broadly triangular, 0.4–0.7 mm long, tips rounded to obtuse, with appressed hairs mostly in the upper half and a single colleter at each side of the base inside; first order axes 0.5–2.5 cm long. Flowers sessile or shortly pedicellate, pedicels 0–1 mm long with central flowers mostly sessile. Calyx green, glabrous outside; tube ca. 0.25 mm long, glabrous and without colleters inside; lobes ovate, 0.2–0.3 mm long, bases not overlapping but closely joining, tips rounded to obtuse, rarely acute. Corolla tube 1.5–2.5 mm long, ca. 0.4 mm in diameter at the base, ca. 1 mm in diameter at the throat, glabrous outside, throat and upper third to half moderately to densely covered with erect hairs inside; lobes oblong, 2–2.5 × 0.75–1 mm, glabrous on both surfaces, tip blunt and emarginate. Stamens completely exserted at anthesis; filaments < 0.5 mm long; anthers 1.3–1.5 mm long. Ovary 0.5–1 mm long, green, glabrous. Style and stigma white, exserted from the corolla tube for 2–5 mm at anthesis; style densely covered with spreading, upwardly directed hairs in upper half; stigma with upper 1.5–2 mm fusiform, longitudinal papillate lines running down for a further 1–1.5 mm. Fruits spherical, 3–3.5 mm in diameter (persistent calyx not included), glabrous, drying dark brown, somewhat glossy and wrinkled when ripe; seeds ca. 2.5 mm in diameter, dark brown.

##### Habitat.

Dry deciduous forest, on sand (white sand and laterite); alt. 0–800 m.

##### Distribution.

Occurring in western Madagascar from 23° to 15° 30'S; recorded in the Atsimo-Andrefana, Menabe, Melaky and Sofia Regions. Fig. 14D.

##### Phenology.

Flowering: December–January; Fruiting: January–March.

##### Vernacular names.

Kerehetika (Martin 8252-SF); masonjohany (dialect Sakalava; Rabarivola 19861-SF); taolakena (dialect Sakalava; Ravelosaona 6592-SF); vahona (Harmelin 10202-RN bis).

##### Vernacular uses.

Wood used by Sakalava against headaches (Razafimandimbison & Bremer 487).

##### Critical notes.

*Pseudocoptosperma menabense* strongly resembles a *Coptosperma* species. Like *Coptosperma*, it has coriaceous, glabrous leaves and terminal, sessile, compact inflorescences with pentamerous white flowers with small-sized corolla tubes, bracteoles, ovaries, calyx tubes and calyx lobes. Furthermore, the fruits have a single ruminate seed. However, *P. menabense* is unique within the group of species currently brought together under the name *Coptosperma* by the combination of the keeled triangular stipules with well-developed awn and the placentation (3 ovules pendulous from a small placenta attached to the upper half of the septum). Some *Coptosperma* species also have three pendulous ovules but their stipules are of a different type, notably, the “bec du canard” type (Capuron 1973). In this case the stipular sheaths are flat with a rounded or obtuse apex, i.e. they are pressed against each other in such a way that their margins meet without overlapping (De Block et al. 2001: fig. 1), whereas the stipules in *P. menabense* are folded around each other (visible only in the youngest stipule pair). Species without the “bec du canard” stipule type usually have ovules (1 to 3) impressed in a large placenta. – Some specimens in the herbarium TEF bear the name *Enterospermum menabense* Capuron, but the species was hitherto not formally described.

##### Preliminary IUCN assessment.

Vulnerable: VU B1ab(i,ii,iii,iv) + 2ab(i,ii,iii,iv). The extent of occurrence (EOO) of *Pseudocoptosperma menabense*, estimated to be 86,558 km^2^, exceeds the limits for the Vulnerable status under sub-criterion B1 but its area of occupancy (AOO), estimated to be 117 km^2^, falls within the limits for the Endangered category under sub-criterion B2. The species occurs in five locations, two of which are in protected areas: Zombitse-Vohibasia National Park and Kirindy Mitea National Park. The species is known from 16 collections, half of which were collected after the year 2000. The major threat for this species is habitat loss by logging for charcoal and timber, burning for grazing and slash-and-burn agriculture both inside and outside the protected areas (Nicoll and Langrand 1989). Hence, based on the above information, the species is listed as Vulnerable.

##### Additional specimens examined.

**MADAGASCAR**. **Mahajanga Province**: Ménabé, forêt de Tsimembo, E d’Ambereny, Antsalova, 29–31 Mar 1966 (fr.), Capuron 24598-SF (BR, P, TEF); Antsalova, Ambereny, 11 Jan 1959 (fr.), Harmelin 10202-RN bis (BR, P, TEF); region of Port Bergé, along RN6, 242 m, 18 Mar 2010 (fr.), De Block, Groeninckx & Rakotonasolo 2354 (BR, G, K, MO, P, S, TAN); forêt Tsimembo, dans la concession Barthe, district Antsalova, 17 Mar 1961 (fr.), Rabarivola 19861-SF (P, TEF); **Toliara Province**: forêt de Jarindrano, rive gauche du haut Fiherenana, E de Maromiandry, Sakaraha, 29 Dec 1961 (fl.), Capuron 20569-SF (BR, P, TEF); forêt d’Andranomena, entre Andranomena et Marofandilia, Morondava, 19 Jan 1962 (fl.), Capuron 20895-SF (BR, P, TEF); Morondava District, forêt de Kirindi, CFPF Morondava (forêt d’Andalandahalo), jardin botanique 2, c. 45 km NE of Morondava, 10 m, 20 Feb 2000 (fr.), Davis, Rakotonasolo & Wilkin 2564 (BR, K, TAN); Kirindi forest, N part - Conoco 7, 16 m, 19 Jan 2007 (fr.), De Block, Rakotonasolo, Groeninckx & Dessein 2187 (BR, MO, P, TAN); Morondava, Kirindi Forest, close to ecotourist camp, 73 m, 20 Jan 2007 (fr.), De Block, Rakotonasolo, Groeninckx & Dessein 2208 (BR, K, MO, P, TAN); Zombitse-Vohibasia National Park, Zombitse, 31 Jan 2007 (fr.), De Block, Rakotonasolo, Groeninckx & Dessein 2257 (BR, K, MO, P, TAN); Lamboukily, 14 km of base camp in Kirindi, 42 m, 20 Jan 2007 (fr.), Groeninckx, Rakotonasolo, Dessein & De Block 102 (BR, MO, P, TAN); Lamboukily, 14 km of base camp in Kirindi, 42 m, 20 Jan 2007 (fr.), Groeninckx, Rakotonasolo, Dessein & De Block 108 (BR, MO, P, TAN); Menabe, 55 km NE of Morondava, route 8 at CPPF, Kirindy forest, 0.25 to 0.5 km NE of principal concession road, 4.5 km E of route 8, block CN4 and CN5, 35 m, 19–20 Mar 1992 (fr.), Noyes, Harder, Rakotobe, Razafindrabeaza & Abraham 1039 (BR, K, MO, P); forêt d’Andranofotsy situé 5 km N du village du même nom, Belo, Tsirihihina, 4 Jan 1953 (fr.), Ravelosaona 6592-SF (BR, TEF); Atsimo-Andrefana, Zombitse-Vohibasia National Park, along Ritik’ala trail, 700 m from the start at the carpark, 750–800 m, 3 Dec. 2003 (fl.), Razafimandimbison & Bremer 487 (UPS).

#### Tulearia

Taxon classificationPlantaeGentianalesRubiaceae

De Block gen. nov.

urn:lsid:ipni.org:names:77178890-1

##### Diagnosis.

Differing from *Paracephaelis sericea* by the presence of shoot dimorphism, the smaller leaves grouped terminally on lateral short-shoots (blades ≤ 3.5 × 1.5 cm vs. 7–21 × 4.5–12 cm in *P. sericea*), the uni- or pauciflorous inflorescences (1–5 vs. 15 to numerous flowers), the trilobate bracts and bracteoles (vs. triangular), the variability in the number of calyx lobes [(4–)5–7 vs. 5], the pollen without supratectal elements (vs. supratectal elements present) and the fruit with 2 ruminate seeds (vs. 4–10 seeds with entire endosperm).

##### Type species.

*Tulearia splendida* De Block.

Shrubs; shoot dimorphism present: vegetative long-shoots with well-developed internodes, reproductive short-shoots with compressed internodes and densely beset with corky stipular remnants; vegetative parts densely pubescent. Leaves grouped terminally on short-shoots, persistent, petiolate with petioles short and canaliculate above; blades < 4 × 1.5 cm, coriaceous; domatia absent; margins strongly revolute. Stipules triangular with short acuminate tip, inside densely covered with appressed hairs all over (hairs visible along the margins from the outside) and with large colleters in the lower half. Inflorescences terminal, sessile, uni- or pauciflorous, cymose with trichotomous branching; all parts (axes, bracts, bracteoles, pedicels) densely pubescent; bracts and bracteoles trilobate. Flowers hermaphroditic, pentamerous, shortly pedicellate; all parts (ovary, calyx, corolla) densely pubescent outside; secondary pollen presentation present. Calyx well-developed, either with short tube and long lobes or with lobes as long as or shorter than tube; lobes (4–)5–7(–8). Corolla white, sericeous outside; tube narrowly cylindrical; lobes contorted to the left in bud and spreading at anthesis. Stamens inserted in the sinuses of the corolla lobes at or somewhat below the level of the throat; filaments short; anthers usually partly included in the corolla tube at anthesis, basifixed, with sagittate base and short sterile apical appendix. Disc annular, fleshy, glabrous. Ovary cup-shaped, bilocular or rarely trilocular; placentation axile, with 3–7 ovules arranged along the periphery of a small placenta attached to the upper half of the septum. Style and stigma white, exserted from the corolla tube at anthesis; stigmatic lobes fused over their entire length except for the very tips, receptive zone on the adaxial surfaces of the free tips and along the lines of fusion of the lobes. Fruits drupaceous, subspherical, pubescent, crowned by the persistent calyx, containing 2 pyrenes; pyrenes crustaceous or stony, hemi-ovoid, formed by the convex outer and flat inner parts of each locule, with central apical protuberance or ridge on the adaxial side, opening along a central longitudinal preformed germination slit present over the entire length on the abaxial and adaxial sides (running through apical ridge or apical protuberance), containing 1 or very rarely 2 seeds; seed hemi-ovoid (or angular in case of 2 seeds per pyrene); hilum ovate, superficial, annulus around hilum absent; exotesta cells parenchymatic and filled with tannins; endotesta consisting of crushed cell layers without crystals; endosperm ruminate. Pollen grains 3-zonocolporate, exine microreticulate to perforate, supratectal elements absent.

A genus of two species, restricted to the dry forest and scrub of southern and southwestern Madagascar, on calcareous soil.

##### Etymology.

The genus is named for its occurrence in the region of Toliara (Tuléar).

##### Key to the species of *Tulearia*

**Table:** 

1	Leaves 10–35 × 6–15 mm, secondary nerves visible; inflorescences with (1–)3(–5) flowers; bracteoles 8–12 mm long; calyx lobes 7.5–10 mm long; calyx tube much shorter than lobes	***T. splendida***
–	Leaves 5–20 × 3–5.5 mm; secondary nerves not visible; inflorescences uniflorous; bracteoles ≤ 3.5 mm long; calyx lobes 1–2 mm long; calyx tube as long as or longer than lobes	***T. capsaintemariensis***

#### Tulearia splendida

Taxon classificationPlantaeGentianalesRubiaceae

De Block sp. nov.

urn:lsid:ipni.org:names:77178892-1

**Figure 3.**
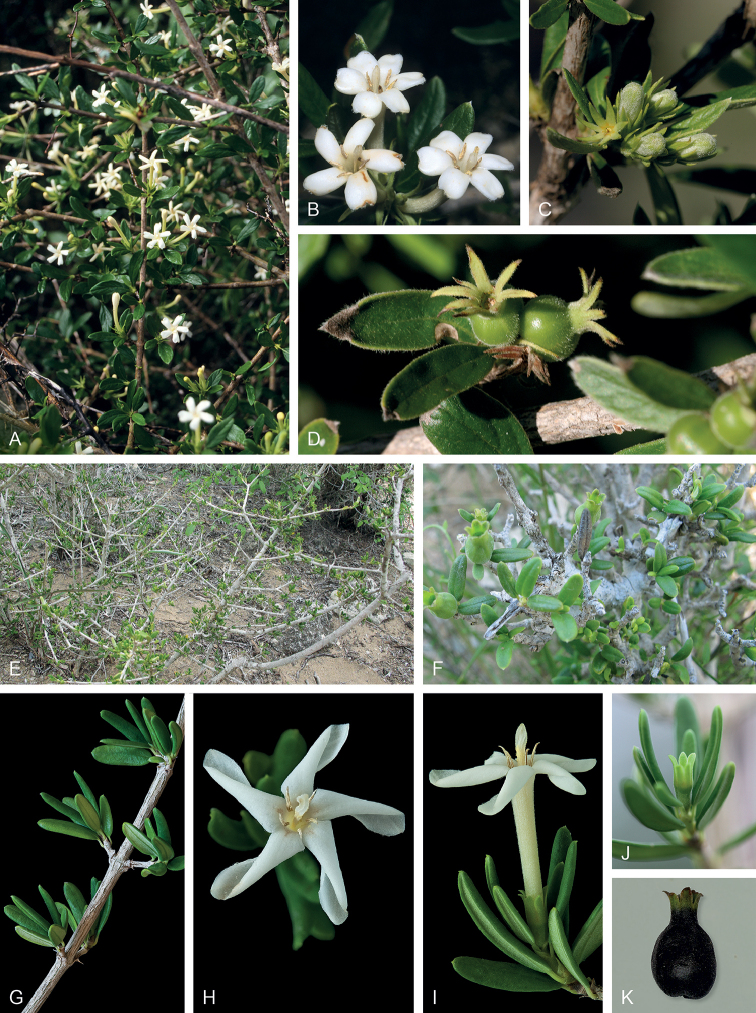
*Tulearia*. **A–D** *Tulearia splendida*: **A** habit **B** flowers **C** young inflorescence showing calyces and flower buds **D** young fruits **E–K** *T. capsaintemariensis*: **E, F** habit **G** branching pattern **H** top view of flower **I** lateral view of flower **J** ovary and calyx **K** fruit. Photographs: P. De Block (**A, B, E, F, J**), S. Dessein (**C, D**), M. Strack Van Schijndel (**G–I**), I. Van der Beeten (**K**).

Figs 3A–D , 11 , 13G–I, L, M

##### Diagnosis.

Differing from *Paracephaelis sericea* by the habit (shrub vs. tree 5–16 m tall), the small leaves (1–3.5 × 0.6–1.5 cm vs. 7–21 × 4.5–12 cm), the pauciflorous inflorescences (1–5 vs. 15 to numerous flowers), the trilobate bracts and bracteoles (versus triangular and vaulted), and the fruit with 2 hemi-ovoid ruminate seeds (vs. 4–10 laterally flattened seeds with entire endosperm).

##### Type.

MADAGASCAR. Toliara Province, La Table, ca. 15 km from Tuléar on RN 7, 4 Jan 1999 (fl.), De Block, Leyman, Dessein, Rakotonasolo & Randriamboavonjy 542 (holotype: BR!; isotypes: BR!, K!, MO!, P!, TAN!).

Shrub to 4 m tall, but usually smaller, densely branched; young shoots brown, moderately to densely covered with erect or spreading hairs, rapidly becoming corky with loss of pubescence; older branches brown, pale brown, fawnish or greyish, corky and somewhat flaking. Leaves elliptic, narrowly elliptic, more rarely obovate, narrowly obovate, ovate or narrowly ovate, 10–35 × 6–15 mm; blades coriaceous, drying brownish-green to brown above, somewhat paler below, densely covered with short erect and more sparsely with long appressed hairs above, lanate but often with hairs more appressed on midrib and secondary nerves below; base cuneate to attenuate; apex rounded and mucronate; midrib and secondary nerves raised on the lower leaf surface; midrib impressed on the upper leaf surface; 4–6(–7) secondary nerves on each side of the midrib. Petioles 1.5–4 mm long, densely covered with appressed or spreading hairs. Stipules caducous, densely covered with appressed hairs outside but rapidly becoming corky and losing the pubescence; sheaths triangular, 2–4 mm long, tips 1–2 mm long. Inflorescences with 1–5 flowers but usually 3-flowered, compact; inflorescence axes, pedicels, bracts and bracteoles densely covered with appressed or spreading to erect hairs; bracts and bracteoles with short petiole-like stalk, trilobate; first order bracts either with central lobe a petiolate leaf similar in size and shape to vegetative leaves and lateral lobes linear, 2–4 mm long, or central lobe narrowly triangular or linear up to 1 cm long and lateral lobes linear, usually much shorter than the central lobe; higher order bracts similar in shape and size to bracteoles; bracteoles subopposite on the pedicel just below the ovary, 8–12 mm long, consisting of a ca. 1 mm long petiole-like stalk, an ca. 1 mm high sheath, a narrowly oblong central lobe, 5–11 × 1–1.25 mm, and 2 linear lateral lobes, 3–5 mm long; bracts and bracteoles densely covered with appressed to erect hairs all over and with scattered colleters in the sheath inside. Flowers sweetly scented, shortly pedicellate, pedicels 1.5–2.5 mm long. Calyx pale green or green, considerably wider than the ovary; tube ca. 1 mm long, densely covered with appressed to spreading hairs outside, densely covered with appressed hairs and with a prominent ring of colleters at the base inside; lobes (4–)5–7(–8), leaf-like or narrowly oblong, somewhat variable in size within a flower, 7.5–10 × 1–2 mm, sometimes linear interstitial lobes up to 5 mm long present or lobes shallowly or profoundly split lengthwise (with two tips), moderately to densely covered with appressed to spreading hairs outside and inside, bases not overlapping but closely joining, tips acute. Corolla sericeous outside; tube 4–30 mm long, ca. 1.5 mm in diameter at the base, 3.5–4.5 mm in diameter at the throat, basal half densely covered with erect hairs inside; lobes oblong or rarely square, 4–8 × 3–6 mm, inner surface glabrous and drying orange or blackish-brown and contrasting with the white pubescence of the corolla tube, margins densely ciliate, tip blunt and emarginate, somewhat assymetrical. Stamens inserted in the sinuses of the corolla lobes at the level of the throat, their bases often included in the corolla tube at anthesis; filaments < 1 mm long; anthers 4–4.5 mm long. Ovary ca. 1.5 mm long, pale green or green, densely covered with erect hairs, often longitudinally ribbed when dry. Placenta attached somewhat above the midde of the septum, with (3–)4–5 ovules arranged along its periphery. Style and stigma white, exserted from the corolla tube for 4–6 mm at anthesis; style sparsely covered with upwardly directed hairs in the upper half (somewhat below the receptive zone of the stigma); stigmatic tips free and spreading for ca. 1 mm, receptive zone ca. 8 mm long, the upper 3–4 mm fusiform, the lower 4–5 mm not widened. Fruits bilobed, 4.5–5 × 5.5–6.5 mm (persistent calyx not included), densely covered with short erect hairs, drying brown or blackish, glossy and somewhat wrinkled when ripe; 2 pyrenes per fruit, crustaceous, with a central apical cuspidate protuberance on the adaxial side; seeds 1(–2) per pyrene, ca. 3.5 × 3–3.5 mm, brown.

##### Habitat.

Open-canopy dry forest, spiny forest, xerophytic thicket, dry scrub, on calcareous soil, both rocky and sandy (e.g. dunes); alt. 0–350 m.

##### Distribution.

Only known from the Atsimo-Andrefana Region in southwestern Madagascar. Fig. 14E.

##### Phenology.

Flowering: December–April; Fruiting: from March onwards.

##### Vernacular names.

Toalanambata (coll. ignot. 31297-SF).

##### Vernacular uses.

Medicine for the eyes (“fanafody des yeux”; Dequaire 27496).

##### Critical note.

*Tulearia splendida* strongly resembles *Paracephaelis* by the general hairiness of the whole plant, the robust sericeous flowers, the well-developed calyx and the ovules arranged on the periphery of the placenta. *Tulearia* differs from *Paracephaelis* by its seeds (2 hemi-ovoid, ruminate seeds in *Tulearia* vs. 4–10 laterally flattened seeds with entire endosperm in *Paracephaelis*) and by its pollen (tectum without supratectal elements vs. tectum with supratectal elements).

##### Preliminary IUCN assessment.

Vulnerable: VU B1ab(i, ii, iii, iv) + 2ab(i, ii, iii, iv). The extent of occurrence (EOO) of *Tulearia splendida* is estimated to be 21,644 km^2^, which exceeds the upper limits for the Vulnerable status but its area of occupancy (AOO), estimated to be 342 km^2^, falls within the limits of the Endangered category. The species is well-collected with both recent and historic specimens. *Tulearia splendida* occurs in eight locations, only one of which lies in a protected area, the Tsimanampetsotsa National Park. The species is threathened by habitat loss as a result of grazing, subsistence farming, logging for timber and charcoal and burning to improve grazing. Based on the above information, the species is assessed as Vulnerable.

##### Critical notes.

Some specimens in the herbarium P were annotated as *Randia tulearensis* Homolle (Perrier de la Bâthie 12816 & 19025). In his unpublished work on the Madagascan Rubiaceae, Capuron (1973) used the name *Enterospermum tulearense* (Homolle) Capuron. But no formal description of the species was hitherto attempted.

##### Additional specimens examined.

**MADAGASCAR**. **Toliara Province**: Tuléar, 16 Dec 1912 (fl.), Afzelius s.n. (P); Tuléar, 16 Dec 1912 (fl.), Afzelius 265 (S); Tuléar, Saint Augustin, 20 Dec 1912 (fl.), Afzelius 268 (S); Tuléar, 16 Dec 1912 (fl.), Afzelius 269 (S); 3 km S of Morombe towards Ampasilava, 14 m, 16 Sep 2006 (fr.), Andriamahay & Rakotoarisoa 1514 (K); Betioky, 350 m, 22 Apr 2004 (fr.), Andriamahay & Rakotoarisoa 750 (K); W d’Ejeda, 15 May 1951 (fr.), Bosser 252 (P); plateau Mahafaly, Ankalirano, W d’Ejeda, Mar 1960 (fl., fr.), Bosser 14302 (P); Tuléar, falaises du Fiherenana, Feb 1962 (fl.), Bosser 15711 (P, TAN); environs du Lac Ihotry, N de Tongobory, 8 Jan 1962 (fl.), Capuron 20713-SF (BR, P, TEF); vers le PK 28 de la route Tuléar-Sakaraha, Dec 1961 (fl.), Capuron & Chauvet 20777-SF (P, TEF); environs de Tuléar, bas de La Table, 4 Mar 1961 (fl.), Chauvet 51 (BR, P, TEF); environs de Tuléar, route de Sarodrano, 11 Mar 1961 (fl.), Chauvet 74 (P); km 22 sur route d’Antananarivo, Tuléar, 9 Nov 1961 (fl.), Chauvet 180 (BR, P, TEF); ancienne route de Sarodrano, Tuléar, 13 Nov 1961 (fl.), Chauvet 198 (BR, P, TEF); route de Sarodrano, 14 Nov 1962 (fl.), Chauvet 362 (P, TEF); La Table, Tuléar, 10 Mar 1963 (fr.), Chauvet 412 (P); Tsimanampetsotsa, Réserve de Manampetsotsa, Lac Folifoetsy, commune Behelony, district Ejeda, 13 Mar 1987 (fr.), coll. ignot. 31297-SF (TEF); vicinity of Tongobory along banks of Onilahy River, 60 m, 14 Feb 1975 (fl.), Croat 31182 (K, P, MO, TAN); dunes on road to Ifaty, 17 m, 2 Feb 2007 (fl.), De Block, Dessein, Groeninckx & Rakotonasolo 2287 (BR, MO, P, TAN, WAG); La Table, 94 m, 2 Feb 2007 (fl.), De Block, Dessein, Groeninckx & Rakotonasolo 2301 (BR, MO, P, TAN, WAG); Ampasimariry, close to Lac Tsimanampetsotsa, Ambola, commune Beheloke, district Tulear II, 14 m, 4 Feb 2007 (fl.), De Block, Dessein, Groeninckx & Rakotonasolo 2311 (BR, MO, P, TAN); Morombe, s.dat. (fr.), Decary 18720 (BR, P); La Table, Tuléar, s.dat. (fl.), Dequaire 27496 (P); environs de Tulear, Ankilibe, 5 Feb 1957 (fl.), Descoings 2326 (TAN); province de Tuléar, s.dat. (fl.), Géay 32 (P); road towards Betioky, 171 m, 3 Feb. 2007 (fr.), Groeninckx, Rakotonasolo, Dessein & De Block 209 (BR, MO, P, TAN, WAG); Lac Tsimanampetsotsa, 5 Feb 2007 (fr.), Groeninckx, Rakotonasolo, Dessein & De Block 216 (BR, G, MO, P, TAN); piste ‘Ajax’, 30 km N de Tuléar, 10 Dec 1968 (fl.), Guillaumet 2288 (BR, P, TAN); Itampolo, Lac d’Itampolo, s.dat. (fr.), Homolle s.n. (P); Tuléar, s.dat. (fr.), Homolle 1566 (P); Lac d’Itampolo, s.dat. (fr.), Homolle O5 (P); gorges de Fiherenana entre Beantsy et Anjamala, 30–300 m, 16–19 Jan 1947 (fl.), Humbert 19889 (BR, P); Manambo, près de la mèr, 20 m, 29–30 Jan 1947 (fl.), Humbert 20089 (BR, P); embouchure de la Menarandra, Bevoalava-Ankazondranto, 1–150 m, 12 Mar 1955 (fr.), Humbert & Capuron 29386 (BR, P); plateau Mahafaly, W de Betioky, 100–300 m, 17–20 Mar 1955 (fr.), Humbert & Capuron 29485 (BR, P); environs de Tuléar, sur la Table, SW flanc, Mar 1960 (fr.), Keraudren 583 (BR, P); environs de Tuléar, bord de mèr, sur les dunes près du village d’Ankilibe, Mar 1960 (fr.), Keraudren 613 (P); plateau calcaire Mahafaly, près du village d’Ankaliano, W d’Ejeda, Mar 1960 (fr.), Keraudren 854-bis (P); Mahafaly, près du village d’Ankaliano, SW de Betioky, Mar 1960 (fr.), Keraudren 856 (P); environs de Tuléar, gorges de Fiherenana, Feb 1962 (fl.), Keraudren 1347 (P); route d’Ampanihy à Androka, 37 km SW d’Ampanihy, colline E de la piste, 230–260 m, 6 Feb 1990 (fr.), Labat, Du Puy & Phillipson 2081 (K, P); Tuléar, La Table, E of town on the road to Antananarivo, 60 m, 28 Feb 1993 (fl.), Luckow 4170 (BR, MO, TAN); 20–30 km N of Tulear on road to Morombe, 5–10 m, 27 Dec 1988 (fl.), Miller & Miller 3791 (K, MO, P, TAN); 14 km SE of Tulear on RN 7, 100 m, 24 Mar 1991 (fr.), Miller & Randrianasolo 6131 (K, MO, P, TAN); route Sahodona-Ampanihy, Mar 1964 (fl.), Morat 637 (P); plateau Mahafaly, Jan 1910 (fl.), Perrier de la Bâthie 9516 (P); environs de Tuléar, Aug 1919 (fl.), Perrier de la Bâthie 12816 (BR, P); environs de Tuléar, Apr. 1933 (fr.), Perrier de la Bâthie 19025 (P); Manampetsa, Apr 1933 (fl.), Perrier de la Bâthie 19124 (P); E of Tulear, around la Table, 100 m, 5 Jan 1989 (fl.), Phillipson 3082 (BR, K, MO, P, TAN, WAG); Réserve de Tsimanampetsotsa, NW corner, 50 m, 11 Jan 1989 (fl., fr.), Phillipson & Rabesihanaka 3156 (K, MO, P, TAN, WAG); 12 km N of Betsiky on road to Tongobory along E facing calcareous escarpment, 300 m, 13 Feb 1990 (fr.), Phillipson 3498 (K, MO, P, TAN); Atsimo-Andrefana, N of Toliara, between Fiherenana and Manombo rivers, Ranobe forest, Belalanda commune, c. 1 km from RN 9 along PK 32 track, 50 m, 15 Mar 2006 (fl.), Phillipson, Ranaivojaona, Andrianjafy & Lubke 5909 (BR, MO); dunes de Befanany, 15 Feb 1921 (fr.), Poisson 149 (P); Réserve naturelle X, Lac Tsimanampetsotsa, canton Soalany, district Betioky, 21 Mar 1953 (fl.), Raodonanahany 5015-RN (BR, P, TAN); Manombo, near PK 32, 11 Dec 2004 (fl.), Rakotonasolo, Smith, Hoffmann, Ralimanana & Sharon 878 (BR, K, MO, P); 1 km before Tongobory on PK 58 from Andranovory, 12 Dec 2004 (fl.), Rakotonasolo, Smith, Hoffmann, Ralimanana & Sharon 885 (BR, K); Tuléar II, Belalanada, Ranobe, forest E of allée des baobabs, 159 m, 26 Jan 2007 (fr.), Ranaivojaona, Manjakahery & Andrianjafy 1699 (K, MO); Andatabo, 18 km S de Tuléar, bord de la RN 7, 50–100 m, 5 Feb 1999 (fr.), Randrianaivo, Ralimanana, Randrianasolo, Andriantiana, Rakotondrajaona & Rahelivololona 325 (BR, K, MO, P); on road to Saint Augustin, 25 km from Tuléar, 17 Feb 1998 (fr.), Razafimandimbison 281 (MO); Betioky, 10 km S of Tongobory, along road to Andranovory, 11 Dec 2003 (fl.), Razafimandimbison 526 (UPS); Betioky, 10 km S of Tongobory, along road to Andranovory, 11 Dec 2003 (fl.), Razafimandimbison 530 (S, UPS); Toliara II, Ranobe, 32 m, 19 Mar 2007 (fr.), Razanatsoa & Manjakahery 369 (BR, MO, P, TAN).

#### Tulearia capsaintemariensis

Taxon classificationPlantaeGentianalesRubiaceae

De Block sp. nov.

urn:lsid:ipni.org:names:77178893-1

Figs 3E–K , 12 , 13D–F, K

##### Diagnosis.

Differing from *T. splendida* by the smaller leaves (5–20 × 3.5–5.5 mm vs. 10–35 × 6–15 mm in *T. splendida*), the secondary nerves which are invisible on both leaf surfaces (vs. visible in *T. splendida*), the uniflorous inflorescences (vs. 1–5 flowers), the shorter bracteoles (up to 3 mm vs. 8–12 mm long), the shorter calyx lobes (1–2 mm vs. 7.5–10 mm long) and the longer calyx tube (1.5–3 mm vs. ca. 1 mm long).

##### Type.

MADAGASCAR. Toliara Province, Fort-Dauphin, road between Faux-Cap and Marovato, 124 m, 3 Apr 2010 (fl., fr.), Groeninckx, De Block & Rakotonasolo 309 (holotype: BR!; isotypes: BR!, K!, MO!, P!, TAN!).

Shrub, 0.5–1.5 m high; young shoots brown, densely covered with spreading hairs, rapidly becoming corky with loss of pubescence; older branches pale brown, fawnish or greyish, corky. Leaves elliptic, narrowly elliptic or rarely broadly elliptic, 5–20 × 3–5.5 mm; blades thickly coriaceous, drying brown to blackish brown and somewhat glossy above, somewhat paler below, densely covered with short erect hairs above, lanate but often with hairs more appressed on midrib below; base obtuse to rounded; apex rounded and mucronate; midrib raised in the basal half on the lower leaf surface, somewhat impressed on the upper leaf surface; secondary nerves invisible on both surfaces. Petioles 1–2 mm long, densely covered with appressed or spreading hairs. Stipules caducous, moderately to densely covered with appressed hairs outside but rapidly becoming corky and losing the pubescence; sheaths triangular, 1.5–2 mm long; tips 0.5–1.25 mm long. Inflorescences uniflorous; bracteoles opposite at the base of the ovary, trilobate or, rarely, reduced to a single lobe; if trilobate, then consisting of a ca. 0.5 mm high basal sheath, 2 linear or narrowly triangular lateral lobes, 0.5–1.5 mm long, and a central lobe, either linear and 1.5–3 mm long or more rarely leaflike (petiole to 2 mm long, blade to 6 × 2 mm, shape identical to that of vegetative leaves), bracteoles moderately covered with appressed or spreading hairs outside, lateral lobes and base of central lobe densely covered with appressed hairs and a few large colleters inside, central lobe higher up round in cross-section and pubescence on adaxial surface identical to that on abaxial surface. Flowers sessile. Calyx green, densely covered with erect or spreading hairs outside, densely covered with appressed hairs inside; tube 1.5–3 mm long, with a ring of colleters at the base inside, the colleters more densely present in the region of the sinuses of the calyx lobes; lobes (4–)5–7, ovate, 1–2 × ca. 1 mm, somewhat keeled when dry, bases not overlapping but closely joining, tips acute to rounded. Corolla sericeous outside; tube 8–10(–18*) mm long, 1.5–2 mm in diameter at the base, 3–4 mm in diameter at the throat, basal half densely covered with erect hairs inside; lobes oblong, 6–7(–12*) × 3.5–4(–5.5*) mm, glabrous inside, margins densely ciliate, tips rounded. Stamens inserted in the sinuses of the corolla lobes ca. 1.5 mm below the level of the throat, only upper half exserted from corolla tube at anthesis; filaments < 1 mm long; anthers ca. 4 mm long. Ovary 1.5–2 mm long, green, densely covered with erect or spreading hairs, faintly ribbed longitudinally when dry. Placenta attached to the upper half of the septum with 5–7 ovules arranged along its periphery. Style and stigma white, 12–14(–22*) mm long, exserted from the corolla tube for 4–5 mm; style densely covered with upwardly-directed spreading hairs in the lower half; stigma fusiform, stigmatic tips free and spreading for ca. 1 mm, receptive zone 6–7(–9*) mm long, widened over the entire length. Fruits bilobed or rarely trilobed, 5–5.5(–7*) × 4.5–5(–7*) mm (persistent calyx not included), densely covered with short erect hairs; when mature, fruit and persistent calyx tube black, calyx lobes remaining green; 2(–3) pyrenes per fruit, stony, with a central vertical ridge apically on the adaxial side; 1 seed per pyrene, ca. 3.5–4 × 3 mm.

##### Habitat.

Open-canopy dry scrub, on calcareous soil, alt. 0–150 m.

##### Distribution.

Only occurring along the coast in the Androy Region in southern Madagascar. Fig. 14F.

##### Phenology.

Flowering & fruiting: April.

##### Critical note.

Measurements indicated with * in the description are from a specimen grown in greenhouse conditions.

##### Preliminary IUCN assesment.

Critically Endangered: CR B1ab(i, ii, iii, iv) + 2ab(i, ii, iii, iv). The extent of occurrence (EOO) of *Tulearia capsaintemariensis* cannot be calculated because only two specimens have ever been collected, but it can be estimated that the EOO is below 100 km^2^. Its area of occupancy (AOO) is 18 km^2^ using a cell width of 3 km but 8 km^2^ using a cell width of 2 km. The species was only discovered in 2010 and occurs in a single location which is not included in a protected area. The main threat to the species is habitat loss as a result of grazing, subsistence farming or land clearing for sisal plantations. Based on the above information, the species is assessed as Critically Endangered.

##### Additional specimens examined.

**MADAGASCAR**. **Toliara Province**: près de Cap Sainte Marie National Park, Valala, 21 m, 4 Apr 2010 (fr.), De Block, Groeninckx & Rakotonasolo 2421 (BR, G, K, MO, P, TAN).

## Discussion

### Phylogenetic analysis

Our analyses confirm many of the results from De Block et al. (2015), notably (1) the monophyly of the Afro-Madagascan clade; 2) the monophyly of the genera *Homollea*, *Robbrechtia* and *Paracephaelis* (including *Homolliella*, represented by its type species *P. sericea*); (3) the polyphyly of the genus *Tarenna*; (4) the breakdown of the Madagascan *Tarenna* species into two highly supported clades; (5) the sister group relationship between the East African monospecific *Tennantia* and the Asian-Pacific species of *Tarenna* (poorly supported); and, (6) the early divergent position of the continental African species *Coptosperma graveolens* and *C. peteri* within the Afro-Madagascan clade II.

As in De Block et al. (2015, clade IV), the backbone of the Afro-Madagascan clade II is poorly supported, but the support for more terminal nodes is high, allowing us to formally recognise at generic level morphologically distinct clades even though their phylogenetic relationships remain unresolved. The phylogeny presented here differs from the 2015 phylogeny in the inclusion of six species endemic to Madagascar. All these taxa fall within clade VII and their respective accessions group together, confirming the hypothesised species concept. Both the *Helictosperma* and *Tulearia* clades, each comprising two species, are highly supported as monophyletic (BPP = 100). The sister-group relationship between *Paracephaelis* and *Homollea*, supported in De Block et al. (2015), has collapsed as *Paracephaelis* is resolved with poor support as sister to clade VII, comprising all the newly included species, the genus *Homollea* and a *Coptosperma* subclade. Our analysis therefore reveals that *Homollea* is more closely related to the *Exallosperma*, *Helictosperma* and *Pseudocoptosperma* clades than to *Paracephaelis* (including *Homolliella*) even though *Homollea* and *Paracephaelis* share highly specific morphological characters, notably the laterally flattened seeds with entire endosperm and a shallow, linear hilum, the two to seven ovules arranged on the periphery of the placenta and the pollen with supratectal microgemmae (De Block et al. 2015; De Block 2018).

Another difference with the analysis by De Block et al. (2015) is the further breakdown of *Coptosperma*. As in De Block et al. (2015), the continental African species *Coptosperma graveolens* and *C. peteri* are early divergent lineages in clade II. However, unlike the 2015 analysis where the rest of *Coptosperma* formed a weakly supported monophyletic group, three distinct *Coptosperma* clades are present here, notably in clades III (*Schizenterospermum*, *Coptosperma littorale, C.* sp. nov. B, *C.* sp. nov. C), VIII (*C. nigrescens*, *C. madagascariense*, *C. supra-axillare*, *C.* sp. nov. D, *C.* sp. nov. E) and IX (*Pseudocoptosperma menabense*). The two main *Coptosperma* clades both contain mostly Madagascan but also some continental African species (*C. littorale*; *C. nigrescens*, *C. supra-axillare*) and they cannot be easily distinguished morphologically. It should also be noted that the backbone of the Afro-Madagascan clade II is unresolved and that the positions of the two main *Coptosperma* clades may change in future analyses. Even the monophyly of *Coptosperma* (after the exclusion of *Pseudocoptosperma menabense*) remains possible. In summary, our results do not support or reject the monophyly of *Coptosperma*; further studies and more data are needed to assess the monophyly of this genus.

The polyphyly of the genus *Tarenna* (De Block et al. 2015) is further confirmed by our analyses. We will not address this issue in this study as our sampling of *Tarenna* is limited. *Tarenna*, as currently delimited, comprises some 200 species, the majority of which is distributed in Asia and the Pacific but with ca. 40 species in continental Africa and ca. 10 species in the Indian Ocean Islands. With the exception of *T. precidantenna* (as outgroup), these African species are not represented in our analyses, which include only seventeen *Tarenna* species in total. With less than 20% of species represented here, we do not feel justified in making taxonomic changes for the genus *Tarenna*. However, a combined molecular-morphological study on the genus, including more taxa, is being undertaken (De Block et al. in prep.).

### Six species in four new genera

The six species studied must be attributed a generic position within the tribe Pavetteae. For this, alternative solutions are possible depending on the amount of morphological variation one allows within generic boundaries. It would be possible to join the six species to existing genera. This could be done at different taxonomical levels. A first solution would be to recognise a single genus for all Madagascan Pavetteae. Clade II is very well supported (BPP = 100), includes all new species and could be recognised at generic level under the name *Coptosperma* (Hooker 1873). We do not favour this solution because the resulting genus would be very variable in all of its characters, both vegetative and reproductive. In fact, this one genus would incorporate almost all morphological variations present in the tribe Pavetteae (e.g. fruits containing 1 to many grains, grains with ruminate or entire endosperm, leaves persistent or deciduous, 1–8 ovules per locule, impressed in, pendulous or arising from the placenta etc.). In order to manage such a diverse genus, subgenera would need to be recognised, notably the same number of subgenera as there are genera now.

A second solution is to join the six species to existing Madagascan genera. The *Exallosperma*, *Helictosperma* and *Pseudocoptosperma* clades could be joined to *Homollea* and the *Tulearia* clade to the *Coptosperma* subclade in clade VIII. We do not favour this solution for the same reason we do not favour a broadly delimited genus *Coptosperma*, notably because it would make the two genera very diverse morphologically. For example, *Homollea* would not only include species with laterally flattened, small lentil-like seeds with entire endosperm, but also species with laterally flattened, large bean-like seeds with thickened surface ridges and entire endosperm (*Exallosperma*), species with small spherical seeds rolled-in on themselves with entire endosperm (*Helictosperma*) and species with small spherical seeds and ruminate endosperm (*Pseudocoptosperma*). The number of pyrenes per fruit would be one or two and the number of seeds would vary between one and ten, while pollen with and without supratectal elements would occur. A similar level of diversity would be present in the taxon combining the subclade of *Coptosperma* and *Tulearia*: species characterised by glabrous vegetative and reproductive organs, flowers with short corolla tubes and small ovaries and calyces and fruits with a single ruminate seed would be combined with densely pubescent species with more robust sericeous flowers (longer corolla, well-developed calyx) and fruits with 2 ruminate seeds. Table 3 gives an overview of the morphological characters for the *Exallosperma*, *Helictosperma*, *Pseudocoptosperma* and *Tulearia* clades as well as for the two remaining subclades in clade VII. While scrutinising this table, it becomes clear that enlarging the genera *Coptosperma* (with the *Tulearia* species) and *Homollea* (with the *Pseudocoptosperma*, *Helictosperma* and *Exallosperma* species) would render these genera rather heterogenous morphologically. As a result, the generic names for Madagascan Pavetteae would no longer have a predictive value with regard to the morphological characters of the species they contain, a situation that should be avoided at all costs.

**Table 3.** Characters of the genera present in clade VII. *number of seeds per fruit: not taking into account cases of aberrant extreme abortion; **ratio calyx tube/calyx lobes is close to 1.

Character/genus	*Coptosperma* (sub-clade in clade VII)	*Homollea*	*Exallosperma*	*Helictosperma*	*Pseudocoptosperma*	*Tulearia*
**Leaves and shoots**						
Shoot dimorphism	absent	absent	present: *Terminalia*-branching	present: *Terminalia*-branching	absent	present
Leaves	coriaceouspersistent	(sub)coriaceouspersistent	papyraceousdeciduous	papyraceousdeciduous	coriaceouspersistent	coriaceouspersistent
Stipule dimorphism	absent	absent	present	present	absent	absent
**Inflorescences**						
Position	terminal, sessile	pseudo-axillary, pedunculate	pseudo-axillary, pedunculate	pseudo-axillary, pedunculate	terminal, sessile	terminal on short-shoots, sessile
Number of flowers	multiflorous	pauciflorous (1–12)	pauciflorous (3–12)	-multiflorous (25–90)^3^-pauciflorous [(1–)5–15(–20)]^4^	multiflorous	-pauciflorous (1–5)^5^-uniflorous^6^
**Flowers**						
Calyx	small, rarely well-developed-tube = lobes**-rarely tube < lobes	well-developedtube < lobes	well-developedtube < lobes	well-developedtube < lobes	smalltube = lobes**	well-developed-tube < lobes^5^-tube ≥ lobes^6^
Length of corolla tube	< 1.5 cm	(0.6–)1.5–3 cm	2.7–3.6 cm	0.5–1.4 cm	0.15–0.25 cm	0.4–3 cm
Outer surface of corolla tube	glabrous	-glabrous-sparsely to densely covered with erect hairs	densely covered with erect hairs	-glabrous-moderately to densely covered with erect hairs	glabrous	sericeous
**Stamens**						
Position at anthesis	completely exserted	partly exserted	partly exserted	completely exserted	completely exserted	partly exserted
Insertion on corolla	in throat	0.5–3 mm below level of throat	ca. 2 mm below level of throat	in throat	in throat	at or ca.1.5 mm below level of throat
**Placentation**						
Number and position of ovules	-1–3 ovules, impressed in placenta-3 collateral ovules pendulous from small placenta^1^	2–7 ovules arising from upper margin of placenta	3–4 ovules arising from upper margin of small placenta	3 ovules arising from upper margin of small placenta	3 ovules pendulous from small placenta	3–7 ovules arranged along the periphery of placenta
Attachment of placenta	middle or upper half of septum	middle or lower half of septum	lower half of septum	lower half of septum	upper half of septum	upper half of septum
**Fruits**						
Number of pyrenes	1	2	2	1	1	2
Number of seeds/fruit*	1	(1–)2–6	2	1	1	2
Texture of pyrene(s)	crustaceous	crustaceous/stony	stony	crustaceous	crustaceous	crustaceous^5^/stony^6^
Opening of pyrene	along the line of fusion of the locules	-absent-along 4 preformed germination slits splitting into 4 valves-along 4 preformed germination slits with the formation of separate stony dispersal units	along a short apical longitudinal preformed germination slit on abaxial and adaxial sides	along 4 preformed longitudinal germination slits; splitting into 4 valves	along the line of fusion of the locules	along a longitudinal preformed germination slit present over the entire length on the abaxial and adaxial sides
**Seeds**						
Seed shape	spherical/ovoidal	laterally compressed (± lentil-shaped)	laterally compressed (bean-shaped)	spherical (rolled-in on itself)	spherical/ovoidal	hemi-sphericalhemi-ovoidal
Hilum	irregular, superficial	linear, superficial	irregularly ovate, superficial	ovate, profound	ovate, superficial	irregular, superficial
Seed surface	smooth (but lines of rumination visible)	smooth	irregular ridges present over the whole surface	smooth	smooth (but lines of rumination visible)	smooth (but lines of rumination visible)
Exotesta cells	parenchymatic	with continuous plate-like thickenings along the outer tangential and the upper parts of the radial walls	with continuous plate-like thickenings along the outer tangential and the upper parts of the radial walls	with continuous plate-like thickenings along the outer tangential and the upper parts of the radial walls	parenchymatic	parenchymatic
Annulus	absent	present	present	present	absent	absent
Endosperm	ruminate	entire	entire	entire	ruminate	ruminate
**Pollen**						
Tectum	perforate to microreticulate	perforate to microreticulate	psilate	perforate to microreticulate	perforate to microreticulate	perforate to microreticulate
Supratectal elements	absent/present^2^	present	absent	absent	absent	absent

^1^ *Coptosperma* sp. D; ^2^ *Coptosperma nigrescens*; ^3^ *Helictosperma malacophylla*; ^4^ *H. poissoniana*; ^5^ *Tulearia splendida*; ^6^ *T. capsaintemariensis*.

We favour the third solution, which is to recognise four new genera, one in clade VIII and three in clade IX. The reason for this is that the six species easily group into four clades which are morphologically distinct from each other and from all other Madagascan Pavetteae genera. We use the criteria of Razafimandimbison et al. (2011) to decide whether these four clades deserve recognition at generic rank or not: (1) they are not nested within other well-defined genera; and (2) they have at least one autapomorphic character or a combination of plesiomorphic characters, allowing them to be recognised easily. Criterion (1) is fulfilled for all four genera in our phylogenetic analysis (Fig. 1). Criterion (2) is also fulfilled. *Exallosperma* differs from all other Pavetteae genera by the fruit consisting of two stony pyrenes, each with a single laterally flattened seed with irregularly distributed ridges on the surface (Fig. 5) and the pollen with psilate tectum (Fig. 9A–D, M). *Pseudocoptosperma* differs from all other representatives in the *Coptosperma* alliance by the combination of two characters: three ovules pendulous from a small placenta (Fig. 10F) and stipules triangular with a strongly developed awn (Fig. 10B). *Helictosperma* is unique by its single spherical seed rolled-in on itself in the shape of a giant pill-millipede (Fig. 8C, D) whereas *Tulearia* is characterised by the combination of robust sericeous corollas (Figs 3I, 11E and 12D, small leaves (Figs 3, 11A, 12A, B), uni- or pauciflorous inflorescences (Figs 3B, C, I, J, 11A, 12A) and fruits with two pyrenes, each with a single ruminate seed (Figs 11G, H, 12F, G).

From a conservation point of view, the description of the new genera is important in order to highlight the existing lineages within the Pavetteae. The *Exallosperma*, *Helictosperma, Pseudocoptosperma* and *Tulearia* lineages are represented by only one or two species. Loss of these species means the loss of unique genetic information only present in their respective lineages (Razafimandimbison et al. 2011).

The description of four new genera to accommodate six species is a valid solution for the Madagascan Pavetteae and is certainly not extravagant when compared to the taxonomic treatment of other Madagascan plant groups. According to Buerki et al. (2013), the Madagascan flora is characterised by endemic genera showing low species diversity, with the ca. 310 endemic Angiosperm genera representing 19% of the generic diversity but comprising only 11.5% of the species present on the island. According to the same authors, one third of all endemic Angiosperm genera in Madagascar are monospecific. The Rubiaceae show a much higher rate of endemism at genus level; they are represented in Madagascar by 91 genera, 29 of which are endemic (De Block 2018), which correlates to 32% of the generic diversity of the Madagascan Rubiaceae. As is the case in the Angiosperms as a whole, the endemic Rubiaceae genera often show low species diversity, accounting for ca. 125 species, or 15.6% of the ca. 800 Rubiaceae species on the island. Also similar to the situation in the Angiosperms as a whole, one third of the endemic genera (10) is monospecific and most of the others are paucispecific (five genera with two species, ten genera with up to seven species). This pattern of a high number of endemic genera with low species diversity is not only found in the Angiosperms. Bauret et al. (2017) present an example in the grammitid ferns: their phylogenetic study revealed more than ten, paucispecific cryptic new lineages in Madagascar and the West Indian Ocean islands.

The description of the four new Pavetteae genera brings the number of Rubiaceae genera in Madagascar to 95, the number of endemic Rubiaceae genera to 33 (36% of the generic diversity), the number of monospecific endemic genera to 12 and the number of endemic genera with two species to seven.

### Habitat, distribution and IUCN assessment

With the exception of *Pseudocoptosperma menabense*, the six species studied here occur on calcareous soil. There is a strong correlation between limestone/calcareous soils and narrow endemism (Du Puy and Moat 1998; Wong et al. 2002), as is also demonstrated by the closely related genus *Homollea* (De Block 2018). *Exallosperma longiflora* and *Tulearia capsaintemariensis* are narrow endemics and they are here assessed as endangered and critically endangered, respectively. *Pseudocoptosperma menabense* and *Tulearia splendida* are assessed as vulnerable. The two *Helictosperma* species have a wider distribution, are known from many collections and are assessed here as near threathened. With the exception of *T. capsaintemariensis*, all new species are represented in at least one protected area. *Exallosperma*, *Helictosperma*, *Pseudocoptosperma* and *Tulearia* occur in southwest, west and north Madagascar in lowland, dry vegation types. *Exallosperma*, *Helictosperma* and *Pseudocoptosperma* occur in closed-canopy dry deciduous or semi-deciduous forest, *Tulearia splendida* in open-canopy dry or spiny forest, xerophytic thicket and dry scrub and *T. capsaintemariensis* in open-canopy dry scrub.

The dry forests in Madagascar are diverse in substrate, vegetation composition and structure. Mostly occurring along the west coast but continuing in southern and northern Madagascar, all dry forests in unprotected areas are under threat of clearing (Waeber et al. 2015). In Madagascar, the dry forests have a similar surface area as the humid forests (ca. 52,500 km^2^), but they are less protected: ca. 29% vs. ca. 48% for humid forests (Waeber et al. 2015). But even protected areas are not free from threat: most have too little funding, too few staff and inadequate infrastructure to effectively ensure conservation of the land surface they are supposed to protect (Nicoll and Langrand 1989; Hannah et al. 2008; Wingen 2011). Madagascan protected areas are under high pressure and suffer encroachment by anthropogenic actions, such as tavy agriculture, fires to improve grazing land, logging for timber or charcoal and traditional and industrial mining (Nicoll and Langrand 1989; Smith 1997; Hartley et al. 2007; Goodman et al. 2008; Waeber et al. 2015). Occurrence in protected areas therefore does not constitute a guarantee for the survival of a species.

While the Madagascan dry forests are generally less rich in species than the humid forests (Waeber et al. 2015), the description of four new genera of the Pavetteae illustrates their importance as a source area of endemic lineages. Twelve other Rubiaceae genera endemic to Madagascar are restricted to the dry forests. Like the four genera described here, they are characterised by low species numbers. Examples are found in the tribes Spermacoceae (*Amphistemon* Groeninckx, 2 species, Groeninckx et al. 2010b: *Lathraeocarpa* Bremek., 2 species, Groeninckx et al. 2009; *Phialiphora* Groeninckx, 2 species, Groeninckx et al. 2010a; *Thamnoldenlandia* Groeninckx, 1 species, Groeninckx et al. 2010b), Pavetteae (*Homollea* Arènes, 5 species, De Block 2018; *Schizenterospermum* Homolle ex Arènes, 4 species, Arènes 1960), Gardenieae (*Melanoxerus* Kainul. & B.Bremer, 1 species, Kainulainen and Bremer 2014), Hedyotideae (*Gomphocalyx* Baker, 1 species, Dessein et al. 2005); Hymenodictyoneae (*Paracorynanthe* Capuron, 2 species, Capuron and Leroy 1978), Mussaendeae (*Landiopsis* Capuron ex Bosser, 1 species, Bosser and Lobreau-Callen 1998); Knoxieae (*Paracarphalea* Razafimandimbison, Ferm, B.Bremer & Kårehed, 3 species, Puff 1988, Ferm et al. 2016) and Octotropideae (*Jovetia* Guédès, 1 species, Guédès 1975). Out of 33 Rubiaceae genera endemic to Madagascar, close to 50% (16 genera) are restricted to the dry forests in western, southern and northern Madagascar.

### Morphological characters

The morphological characters of the four genera are compared here with the characters of the Pavetteae as a whole but with a focus on the groups in clade VII of the phylogenetic tree. The four new genera each have a different fruit, pyrene and seed type and also the placentation is variable.

The four new genera exhibit marked adaptations to their dry habitats in their habit and in their vegetative and reproductive organs. Examples are the pubescent vegetative and reproductive parts, the small (*Tulearia*) or deciduous leaves (*Exallosperma* and *Helictosperma*) as well as the shoot dimorphism and the terminal grouping of leaves. Shoot dimorphism was shown to be strongly correlated with deciduousness (Dörken 2012), which is typical for dry habitats, and this is also the case in *Exallosperma* and *Helictosperma*. Further adaptations to drought are found in the length variation of the corolla tube in certain species (larger flowers demand more water) and certain fruit and seed characters, such as ruminate seeds or pyrenes with opening mechanisms.

**Figure 4.**
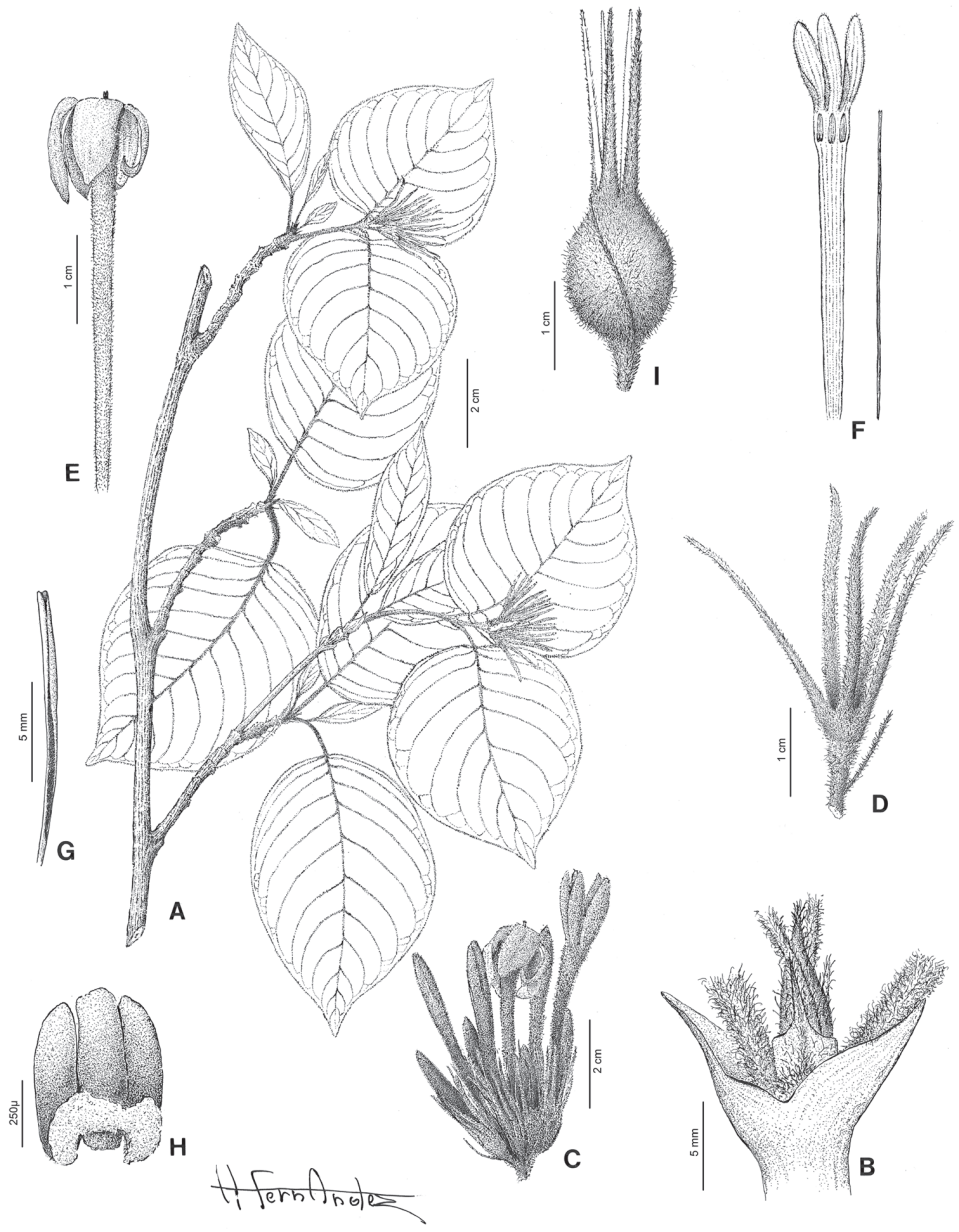
*Exallosperma longiflora*. **A** habit **B** stipules **C** inflorescence **D** bracteole, ovary and calyx **E** corolla and stigma **F** longitudinally opened flower, showing the position of stamens and style **G** stigma **H** placenta and ovules, abaxial view **I** fruit (with bracteole). **A–G** Capuron 24425-SF **H** De Block et al. 1132 **I** Capuron 24663-SF.

**Habit**. Plants of the four new genera are small to medium-sized shrubs or small trees. Shoot dimorphism, i.e. an architecture of long-shoots and short-shoots, occurs in *Tulearia*, in which leaves and inflorescences are grouped terminally on lateral short-shoots (Figs 3G, 11A, 12A). A particular type of shoot dimorphism is found in *Exallosperma* and *Helictosperma*. In these genera, the lateral branches are modular, sympodial and plagiotropic by apposition. Each module of the plagiotropic branches consists of a long first internode which is horizontal in orientation. Distally the internodes are progressively shorter and the apical meristem is reorientated and becomes erect, producing an erect short-shoot (Figs 2A, D, 4A, 7A). Upon the reorientation of the apical meristem, an axillary meristem takes over the further extension of the branch. Leaves and inflorescences are grouped terminally on these erect short-shoots. This type of branching is referred to as *Terminalia*-branching and the trees exhibiting it are often called pagoda trees (Corner 1952: 30). This growth form agrees with Fagerlind’s model (Hallé et al. 1978).

According to Fisher and Honda (1979), trees with *Terminalia*-branching pattern occur in many different habitats, such as evergreen rain forests, seasonally dry forests, coastal or swamp areas. In the Madagascan Pavetteae, however, this branching pattern is only found in species from dry deciduous forests. This is the case for *Exallosperma* and *Helictosperma* and also for the genus *Schizenterospermum*.

According to Hallé et al. (1978), *Terminalia*-branching is typical for relatively small trees restricted to the forest understorey and designed to produce small numbers of seeds at frequent intervals. Fisher and Honda (1979) stated that *Terminalia*-branching ensures efficient light interception by maximising the effective leaf area (EA, the leaf surface directly exposed to sunlight), thereby enhancing the success of plants with this branching pattern. Ashton (1978) noted the success of seedlings with *Terminalia*-branching: they can rapidly increase leaf surface, exposing it in a dense layer above other seedlings.

In *Exallosperma*, *Helictosperma* and *Tulearia*, the short-shoots have little or no internode stem elongation and are covered entirely with stipular remnants. On the short-shoots in *Exallosperma* and *Helictosperma*, vegetative and reproductive nodes alternate. The stipules are dimorphic; their size and shape differ depending on the type of node (see below).

**Leaves**. Leaf arrangement is decussate as is the case in most Pavetteae and Rubiaceae. With the exception of *Pseudocoptosperma*, in the new genera, the leaves are grouped terminally on short-shoots (Figs 2A, D, 3G, 4A, 7A, 11A, 12A). The leaves are of moderate size (0.5–15 × 0.3–9.5 cm) and petiolate, the petioles short in *Pseudocoptosperma* and *Tulearia* (1–6 mm long) and longer in *Exallosperma* and *Helictosperma* (5–45 mm long). Leaves are coriacous and glabrous in *Pseudocoptosperma*, as is also the case in *Coptosperma*. They are strongly reduced in size (0.5–3.5 × 0.3–1.5 cm), coriacous to almost succulent and densely pubescent on both leaf surfaces in *Tulearia*. In *Helictosperma* and *Exallosperma*, the leaves are papyraceous, pubescent (except sometimes in *H. poissoniana*), deciduous and often immature at the time of flowering. In these two genera, the leaf bases are rounded, subcordate, cordate, unequal, truncate or obtuse whereas they are cuneate to attenuate in *Pseudocoptosperma* and *T. splendida* and obtuse to rounded in *T. capsaintemariensis*. Domatia are present in *Exallosperma* and *Helictosperma* (hair tuft or ciliate pit domatia). They are absent in *Pseudocoptosperma* (lower leaf surface glabrous) and *Tulearia* (lower leaf surface lanate).

**Figure 5.**
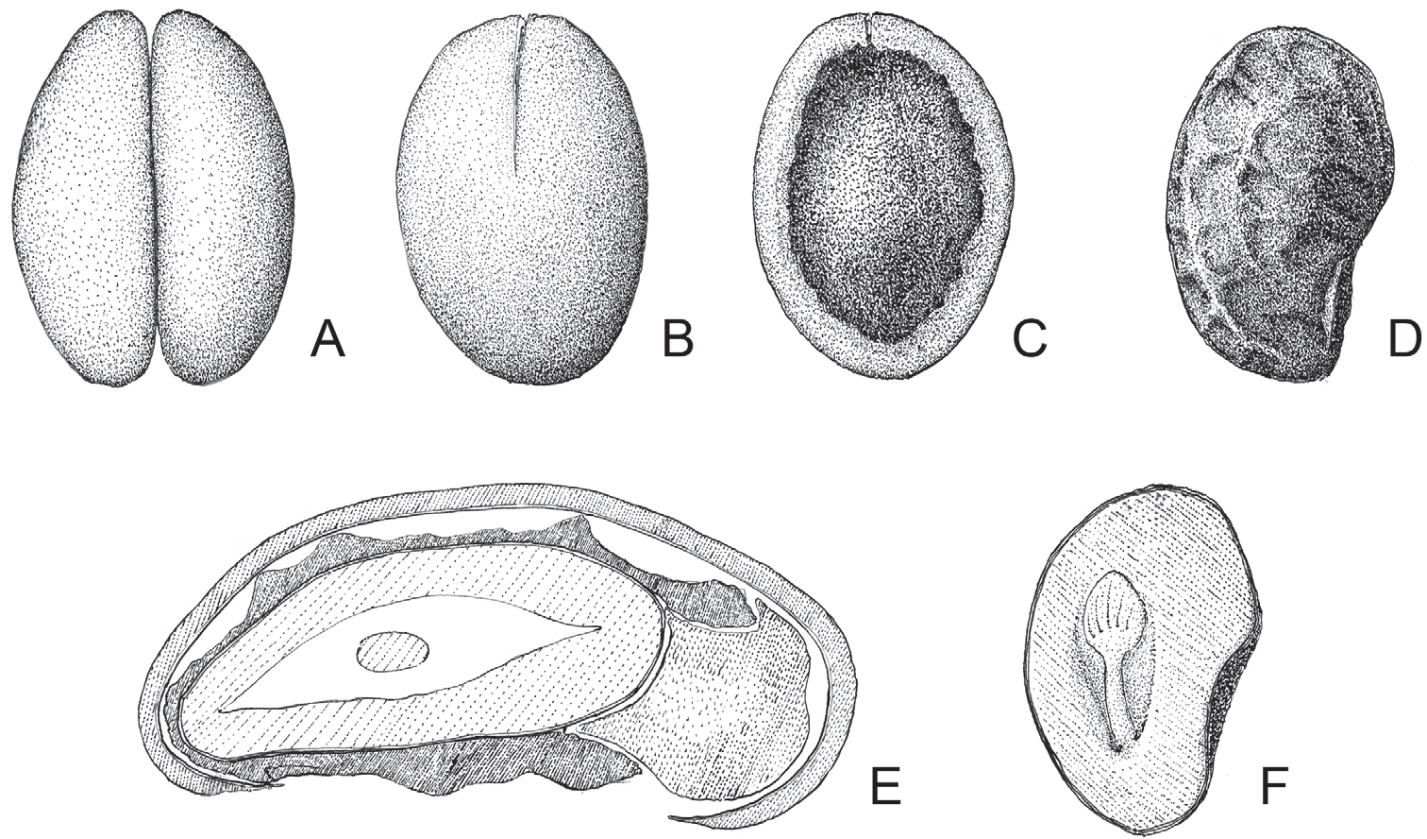
*Exallosperma longiflora*: pyrene and seed. **A** fruit with exocarp and mesocarp removed, showing two pyrenes **B** abaxial view of pyrene, showing apical preformed germination slit **C** adaxial view of pyrene, showing apical preformed germination slit and open centre **D** lateral view of seed, showing irregular ridges on the seed surface **E** cross-section through pyrene and seed, showing the adaxial opening of the pyrene, the entire endosperm and the irregular ridges formed by strongly elongated exotesta cells **F** longitudinal section of seed, showing the embryo position. **A–F** Capuron 24663-SF.

**Stipules**. In the four new genera, stipules rapidly become corky, thereby losing their outside pubescence and are caducous. In *Exallosperma*, *Helictosperma* and *Pseudocoptosperma*, as well as in *Homollea* and in *Coptosperma*, the inner surface of the stipules is glabrous except for a basal row of colleters sometimes interspaced with hairs. In *Tulearia*, the inner surface of the stipules is densely covered with appressed hairs all over and with large colleters in the lower half.

In *Exallosperma* and *Helictosperma*, stipule dimorphism occurs (Figs 4B, 7B). Both genera have the *Terminalia*-branching pattern with leaves and inflorescences grouped terminally on erect short-shoots. On these short-shoots, vegetative and reproductive nodes alternate and each node type has a typical stipule type. The stipules of vegetative nodes are less robust than those of reproductive nodes. They consist of truncate or triangular sheaths forming a cone and each sheath is topped by a needle-like awn. The sheaths vary in length between 1.5 and 5 mm, the awns between 1.5 and 6 mm. In reproductive nodes, the stipules are ovate sheaths with acute or shortly acuminate tips. Their length varies between 4 and 8 mm. Within the Pavetteae and even the Rubiaceae, this type of stipule dimorphism is unknown outside of *Exallosperma* and *Helictosperma*.

**Inflorescences**. In the Pavetteae, the inflorescences are trichotomously branched and their position is usually terminal on leafy lateral branches. This is also the case in *Pseudocoptosperma* and *Coptosperma* (with the exception of *C. supra-axillare*). In *Tulearia*, the inflorescences are also terminal but on lateral short-shoots whereas the inflorescences in *Exallosperma* and *Helictosperma* seem terminal on vertical short-shoots but are in fact pseudo-axillary. These pseudo-axillary inflorescences start out in a terminal position but a lateral bud takes over the vegetative growth after the formation of the inflorescence, thereby pushing it aside into an axillary position. Pseudo-axillary inflorescences are also found in the genus *Homollea*. In the four new genera, there is a correlation between the position of the inflorescence and whether inflorescences are sessile or pedunculate. Terminal inflorescences are sessile (*Pseudocoptosperma*, *Tulearia* and *Coptosperma*), while pseudo-axillary inflorescences are pedunculate (*Exallosperma*, *Helictosperma* and *Homollea*). The inflorescences in the Pavetteae are usually multiflorous, as is also the case in *Pseudocoptosperma*, *Helictosperma malacophylla* and *Coptosperma*. They are, however, pauciflorous in *Exallosperma*, *H. poissoniana*, *Tulearia splendida* and *Homollea* and uniflorous in *T. capsaintemariensis*. In *Exallosperma longiflora*, anthesis is asynchronous within inflorescences, a rare character within the Pavetteae.

**Figure 6.**
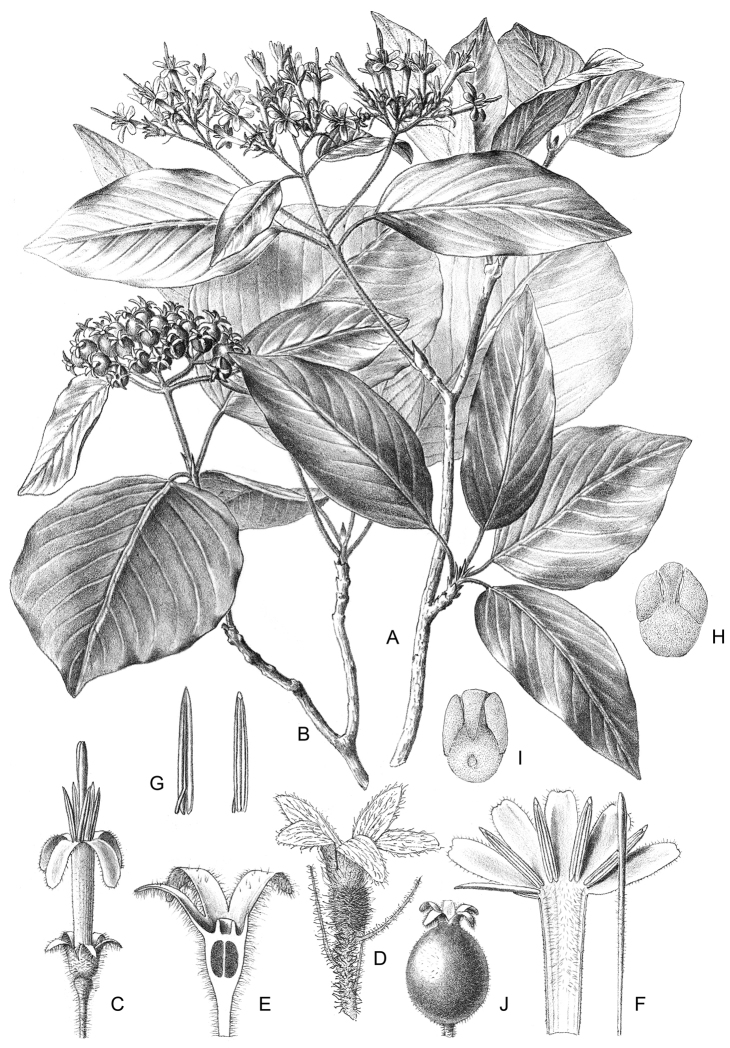
*Helictosperma malacophylla*. A, flowering branch **B** fruiting branch **C** flower **D** bract, bracteole, ovary and calyx **E** longitudinal section through ovary and calyx **F** longitudinally opened corolla showing the position of stamens and style **G** stamens **H** placenta and ovules, abaxial view **I** placenta and ovules, adaxial view **J** fruit. **A–C, E–G, J** reproduced or adapted from Drake del Castillo (1897: Pl. 422) **D** De Block et al. 534 **H, I** De Block et al. 797.

**Figure 7.**
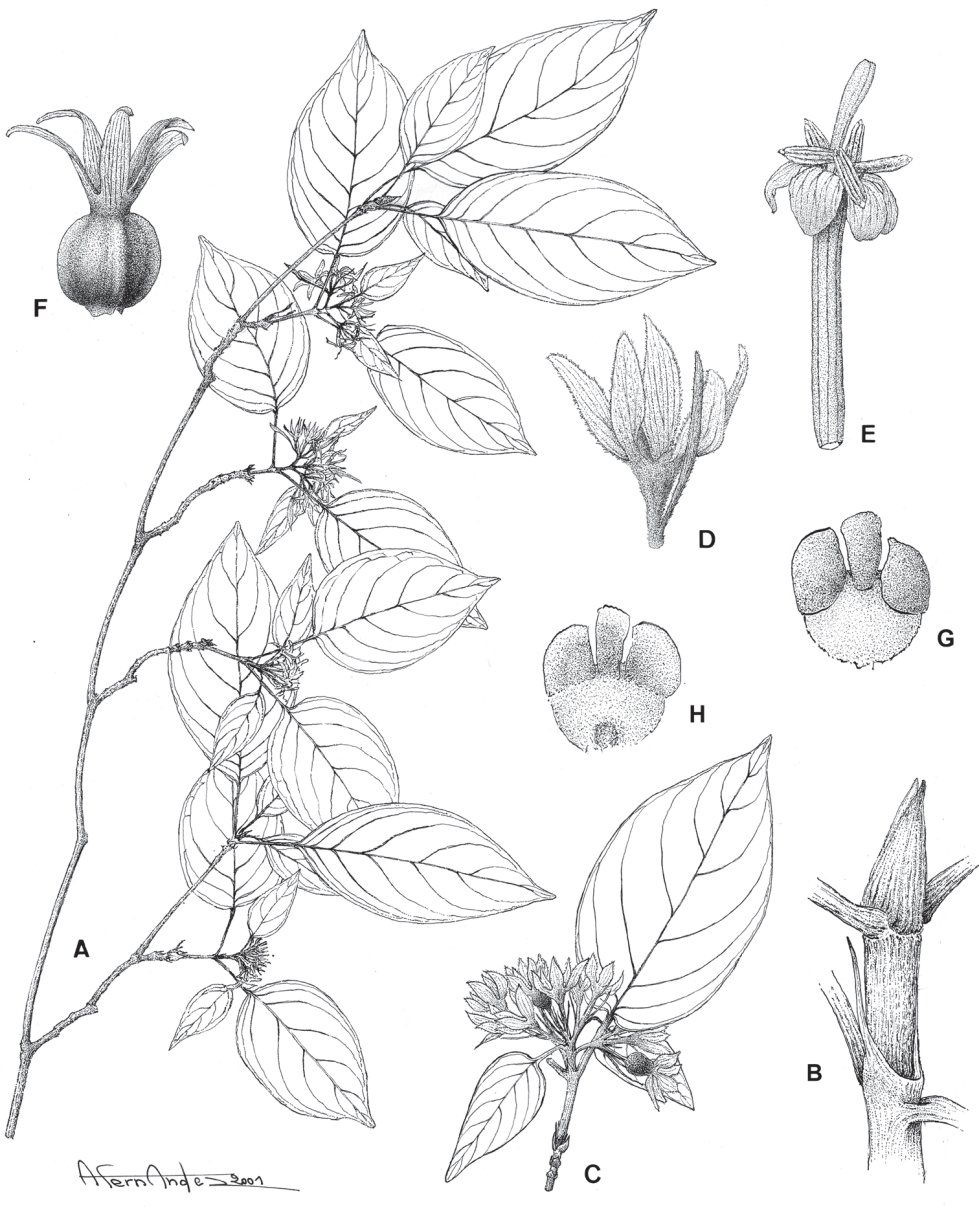
*Helictosperma poissoniana*. **A** flowering branch **B** dimorphic stipules of vegetative and reproductive nodes **C** short-shoot bearing young infructescence **D** bracteole, ovary and calyx **E** corolla, style, stigma and anthers **F** fruit **G** placenta and ovules, abaxial view **H** placenta and ovules, adaxial view. **A, C** De Block & Rakotonasolo 797 **B** De Block 910 **D, E** Jongkind et al. 3415 **F** Randriamiera 8795-RN **G, H** Jongkind et al. 3258.

In the four new genera, bracts and bracteoles are well-developed in species with well-developed calyces [*Exallosperma* (Fig. 4D, I); *Helictosperma* (Figs 6D, 7D) and Tulearia (Figs 11C, D, 12C)] and reduced in species with small calyces [*Pseudocoptosperma* (Fig. 10C, D)]. The same is true for several other Pavetteae genera, e.g. *Coptosperma* (short bracts, bracteoles and calyx lobes) and *Homollea* (well-developed bracts, bracteoles and calyx lobes). The first order bracts at the base of the inflorescences are usually similar to a vegetative node: the stipular parts resemble the stipules and the foliar parts are well-developed leaves, either identical in size and shape to the vegetative leaves or somewhat smaller (e.g. *Helictosperma*) or similar in size but differing in shape (e.g. broadly ovate to orbiculate vs. ovate, elliptic or broadly ovate in *Exallosperma*). Second order bracts of the central axis are often similar to the first order bracts but with the leaves considerably reduced in size, those of the lateral axes being usually similar to higher order bracts. Higher order bracts are reduced with the stipular parts absent and the foliar parts similarly shaped as the bracteoles. Size and shape of the bracteoles varies considerably within the four new genera. They are trilobate in *Tulearia*, linear in *Helictosperma* and *Exallosperma* and broadly triangular in *Pseudocoptosperma*.

In *Helictosperma* inflorescences, the first order branching is often shifted up to 1 cm above the position of the first order bracts (Fig. 6A). Bracts are sometimes adnate to the axes they support for up to 5 mm.

**Calyx**. In three of the four new genera, the calyx is well-developed. This is not the case for *Pseudocoptosperma* (Fig. 10D) in which both calyx tube and lobes are ca. 0.25 mm long. Such small calyces are also common in the genus *Coptosperma*. In the other three new genera, the calyx tube is up to 1 mm long and shorter than the calyx lobes except for *Tulearia capsaintemariensis* (Fig. 12C) where the calyx lobes are shorter than the tube (tube 1.5–3 mm and lobes 1–2 mm long). The longest calyx lobes are found in *Exallosperma longiflora* (12–16 mm long; Fig. 4D, I). The long and narrow calyx lobes of *Exallosperma* are reminescent of the calyx lobes in the genus *Homollea*. *Tulearia* shows variation in the number of calyx lobes, with (4–)5–7(–8) lobes per calyx. Additionally, linear interstitial lobes up to 5 mm long are sometimes present and calyx lobes may be shallowly or profoundly split lengthwise in *T. splendida* (Fig. 3D).

In *Exallosperma*, *Pseudocoptosperma* and *Helictosperma poissoniana*, the calyx tube is glabrous inside and no colleters are present. In *H. malacophylla*, a sparse ring of hairs is present at the base of the calyx tube inside but colleters are missing. Colleters are also absent in the calyx tube in the genera *Coptosperma* and *Homollea*. Only in *Tulearia*, the calyx tube is densely covered with appressed hairs inside and a conspicuous basal ring of colleters is present.

**Corolla**. The four new genera have the typical hypocrateriformous Pavetteae flowers although the corolla tube widens slightly at the level of the throat especially in those species with partly included anthers. Usually, the corolla tube is (much) longer than the corolla lobes in flowers of the Pavetteae. This is also the case in *Exallosperma*, *Helictosperma* and *Tulearia*, but not in *Pseudocoptosperma* in which the corolla lobes are as long as or even somewhat longer than the corolla tube. *Pseudocoptosperma menabense* has atypically small flowers with the total length (corolla tube + lobes) up to 5 mm long. In some species the length of the corolla tube varies considerably, for example, in *T. splendida* in which the corolla tube varies in length between 4 and 30 mm. Length variation also occurs in *H. poissoniana* (corolla tube 5–14 mm long) and *T. capsaintemariensis* (corolla tube 8–10 mm long but 18 mm long in greenhouse conditions). This variation in flower size is probably the result of drought stress. Floral traits have been shown to be influenced by abiotic factors such as the availability of water (Galen 1999; Caroll et al. 2001), with larger flowers requiring more water. The two *Tulearia* species occur in the driest habitats, being part of open-canopy dry forest and scrub vegetation whereas *Exallosperma*, *Helictosperma* and *Pseudocoptosperma* species occur in closed-canopy dry forest.

In *Tulearia*, the corolla is sericeous outside, a situation which is also found in species of the genus *Paracephaelis*. Pubescent corolla tubes are also found in *Exallosperma*, *Helictosperma malacophylla*, in part of the *H. poissoniana* specimens and in some *Homollea* species. The corolla of *Pseudocoptosperma menabense* is glabrous outside, which is also the case in *Coptosperma*.

**Androecium**. The four new genera possess the typical anthers of the Pavetteae: linear, sagittate at the base and with the connective continuing into a short sterile apical appendix (Fig. 6G). The stamens are inserted in the sinuses of the corolla lobes at or somewhat below the level of the throat. Insertion at the level of the throat is the common situation in the tribe Pavetteae and usually results in complete exsertion of the anthers at anthesis, as is the case in *Helictosperma* (Fig. 7E), *Pseudocoptosperma* (Fig. 10E) as well as in *Coptosperma*. In *Exallosperma* (Fig. 4F), the stamens are inserted ca. 2 mm below the level of the throat and included in the corolla tube for most of their length at anthesis. In *Tulearia capsaintemariensis* (Figs 3I, 12D), the situation is similar to that in *E. longiflora* whereas, in *T. splendida* (Figs 3B, 11E), the stamens are inserted at the level of the throat but their bases are often included in the corolla tube at anthesis. Stamens inserted below the level of the throat and partly included at anthesis are rare within the Pavetteae but are found in, for example, *Homollea* and the continental African genus *Leptactina*. Filaments are short (<1.5 mm long) in *Helictosperma*, *Pseudocoptosperma* and *Tulearia* and absent in *Exallosperma longiflora*, the anthers of which are sessile.

**Gynoecium**. The four new genera have small cupular bilocular ovaries and axile placentation, which is the typical situation in the Pavetteae. In many Pavetteae such as, for example, most *Coptosperma* species and the genera *Tarenna* and *Pavetta*, the ovules are impressed in the placental tissue. This is not the case in the four new genera which have small placentas with ovules at their periphery. Ovule number is very variable in the Pavetteae and varies from a single ovule per locule in *Pavetta* to up to ca. 100 ovules per locule in *Leptactina*, but the four new genera are pauciovulate with 3 ovules per placenta in *Helictosperma* (Figs 6H, I, 7G, H) and *Pseudocoptosperma* (Fig. 10F), 3–4 in *Exallosperma* (Fig. 4H) and 2–7 in *Tulearia* (Fig. 12H, I). These numbers are similar to those in closely related genera, for example, 1–3 ovules per placenta in *Coptosperma* and 2–7 in *Homollea*. Further variation is found in the attachment of the placenta: to the basal half of the septum in *Homollea*, *Exallosperma* and *Helictosperma*, to the upper half of the septum in *Coptosperma*, *Pseudocoptosperma* and *Tulearia*. In the genera with basally attached placentas, the ovules arise from the upper margin of the placenta, whereas they are impressed in the placenta (*Coptosperma*), pendulous from the sides or base of the placenta (*Pseudocoptosperma*; *Coptosperma* sp. nov. D) or arranged along the periphery of the placenta (*Tulearia*) in the other genera.

The four new genera show secondary pollen presentation (Puff et al. 1996), which is typical for the Pavetteae. The flowers are proterandrous with the anthers opening and depositing the pollen on a receptaculum pollinis before the flower opens. The receptaculum pollinis can be a part of the style or the stigma or both. When the flower opens, the pollen is presented to flower visitors (functionally male stage). Only at a later stage the receptive zones on the stigma become active (functionally female stage). In the four new genera, the style and stigma are exserted from the corolla tube for 2–10 mm at anthesis. The stigmatic lobes are permanently fused over their entire length (*Pseudocoptosperma*) or only their tips are free as in *Exallosperma*, *Helictosperma* and *Tulearia*. The receptive zones are found on the adaxial surfaces of the free tips and on the lateral sides of the fused stigmatic lobes, visible as two longitudinal papillate zones along the lines of fusion. In most of the species studied here, only the upper part of the stigmatic lobes is thickened, for example, the upper 4–5 mm in *Helictosperma*, the upper 2–3 mm in *Pseudocoptosperma* and the upper 3–4 mm in *Tulearia splendida* are fusiform. However, the receptive zones continue further down along the lines of fusion for 2–4 mm in *Helictosperma*, 1–1.5 mm in *Pseudocoptosperma* and 4–5 mm in *T. splendida*. In *T. capsaintemariensis*, the entire receptive zone is widened and, in *Exallosperma longiflora*, the entire 14–16 mm long fused stigmatic lobes are ± unthickened. A considerable part of the receptive zone is therefore situated below the thickened zone of the stigma and may even be included within the corolla tube at anthesis.

In *Exallosperma longiflora*, the anthers are positioned somewhat below the tips of the stigma in mature buds. Pollen is deposited on the fused stigmatic lobes below the tips (receptaculum pollinis). In this region, the lines of fusion between the stigmatic lobes are visible but papillae are absent. They are only present above (adaxial surfaces of the free stigmatic lobes) and below (lines of fusion of the stigmatic lobes) the zone where the pollen is deposited (Fig. 4G). As a result, there is spatial separation between the pollen-receptive and pollen-presenting surfaces of the stigma. This spatial separation also means that the majority of the pollen receptive zones are included in the corolla tube at anthesis (for a length of 8–10 mm). The papillate zones become wider further down the corolla tube and, at their bases, almost the entire circumference of the stigma is receptive. Furthermore, the receptive longitudinal grooves do not run straight down but slowly circle the stigma. As a result, an insect with a long, pollen-covered proboscis reaching into the corolla tube would always come into contact with the receptive zone. A similar displacement of the receptive zones towards the lower regions of the stigma has been reported for species of two other Pavetteae genera, notably the continental African *Nichallea* and *Rutidea* (De Block and Igersheim 2001).

**Fruits and pyrenes**. The fruits of the four new genera are small drupes crowned by a persistent calyx as is typical in the Pavetteae. The fruits are pubescent in *Exallosperma*, *Helictosperma* (not always in *H. poissoniana*) and *Tulearia* but glabrous in *Pseudocoptosperma*. The fruits are spherical or ovoidal. Their colour at maturity is poorly known as most fruiting specimens were recorded as having green fruits. Fruits becoming brown are mentioned for *Tulearia splendida* (Phillipson 3498). For *Helictosperma poissoniana*, fruits are mentioned as brown (Davis et al. 3122) or white (De Block et al. 1042 & 1242). Only for *T. capsaintemariensis*, unequivocal fruit colours are known since fructification was observed under greenhouse conditions. The fruits are shiny black at maturity with the persistent calyx tube black as well but with the calyx lobes remaining green (Fig. 3K).

**Figure 8.**
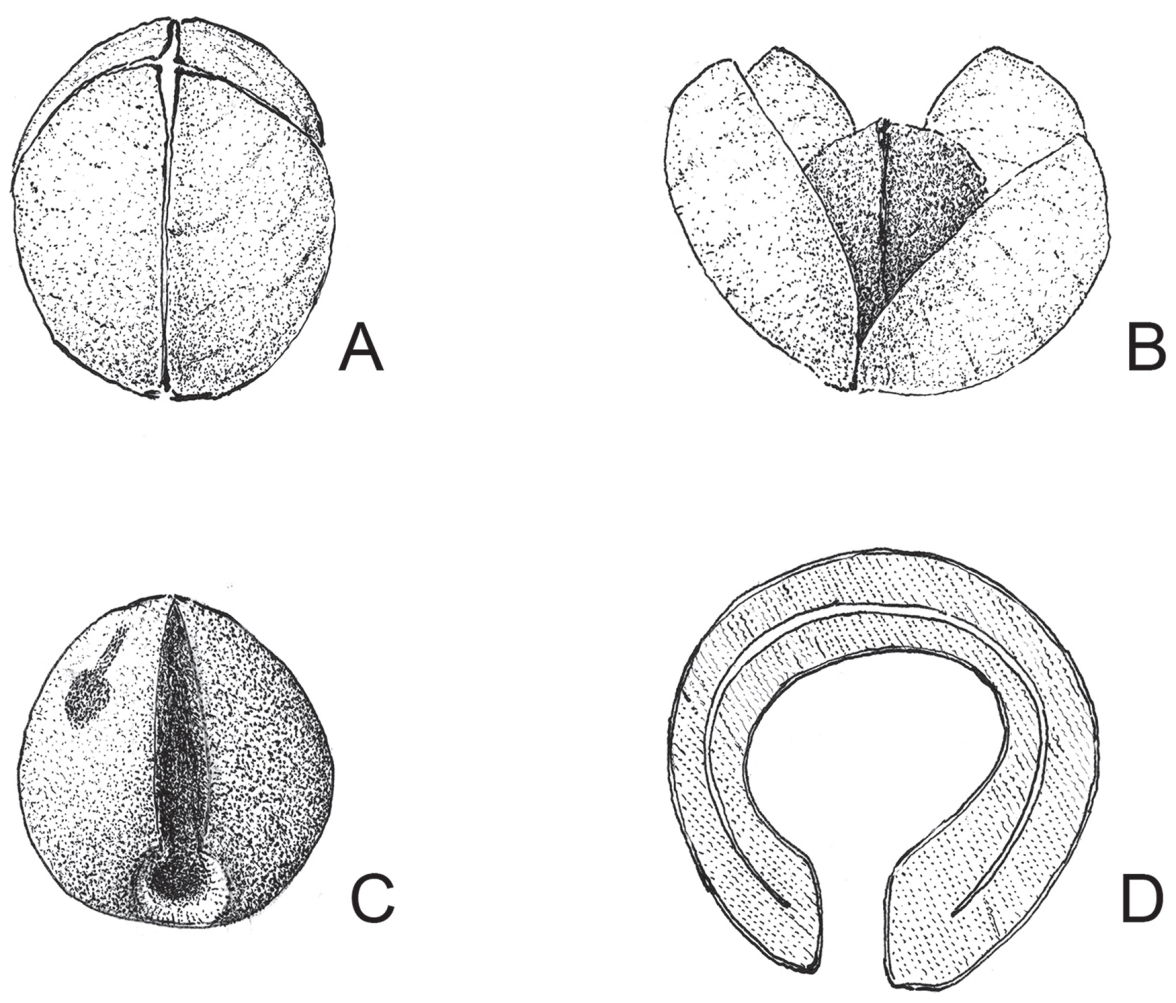
*Helictosperma malacophylla*: pyrene and seed. **A** pyrene showing four preformed germination slits, lateral view **B** pyrene falling apart into four valves along preformed germination slits, lateral view **C** seed, adaxial view, with embryo position indicated **D** transverse section through seed. **A–D** coll. ignot. 19146-SF.

The fruits have a thin exocarp and mesocarp. The endocarp forms one or two pyrenes. Two pyrenes occur in *Exallosperma* (Fig. 5A) and *Tulearia*, a single pyrene in *Helictosperma* (Fig. 8A) and *Pseudocoptosperma* (Fig. 10H). Pyrenes contain a single seed although very rarely two seeds were encounted in pyrenes of *T. splendida*. Pyrenes are formed differently depending on their number within a fruit. In case of a single pyrene, it is formed by the convex outer parts of the two locules within the ovary without the incorporation of the flat inner parts of the locules, i.e. the septum, which remains membraneous and is pushed to the side by the development of the single seed. In case of two pyrenes, they are formed by the convex outer as well as the flat inner parts of the locules: the septum is an integral part of each of the two pyrenes. Clearly, the shape of the pyrene is also dependent on the number of pyrenes per fruit: subspherical or subovoidal in case of a single pyrene and hemispherical or hemi-ovoidal in case of two pyrenes per fruit.

**Figure 9.**
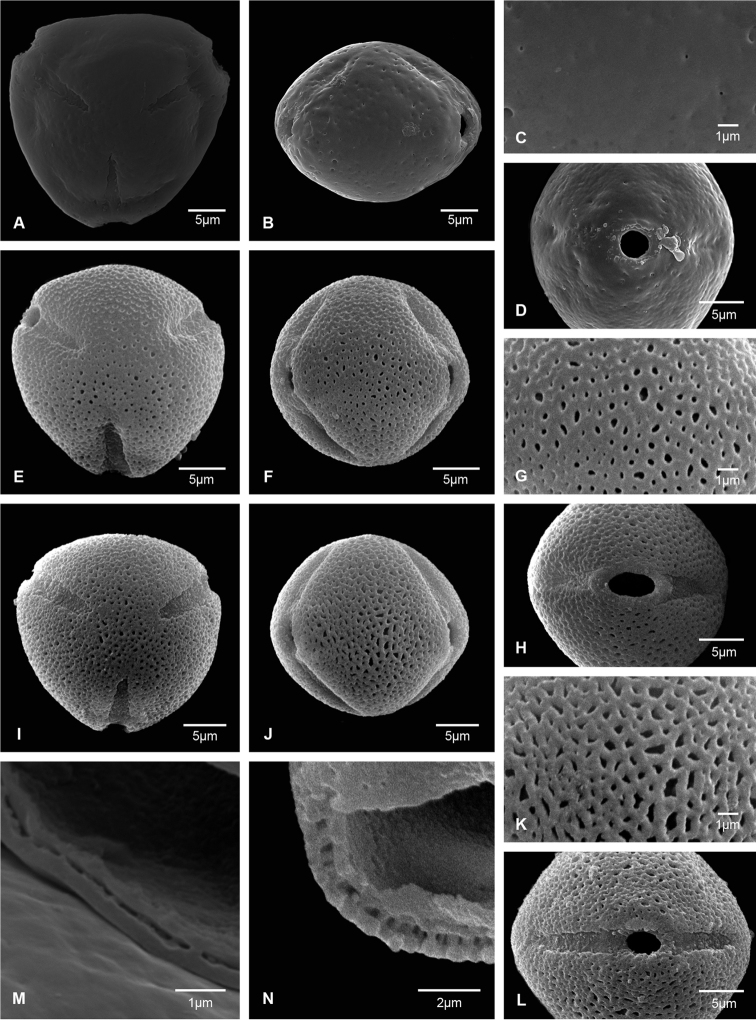
Pollen of *Exallosperma* and *Helictosperma*. **A–D, M** *Exallosperma longiflora* **E–H, N** *Helictosperma malacophylla* **I–L** *H. poissoniana*. **A, E, I** polar view **B, F, J** equatorial view **C, G, K** mesocolpium **D, H, L** ectoaperture **M, N** pollen grain wall. **A, M** Nusbaumer & Ranirison 1992 **B–D** De Block et al. 1132 **E–H, N** Phillipson 3068 **I–L** Leandri 573.

**Figure 10.**
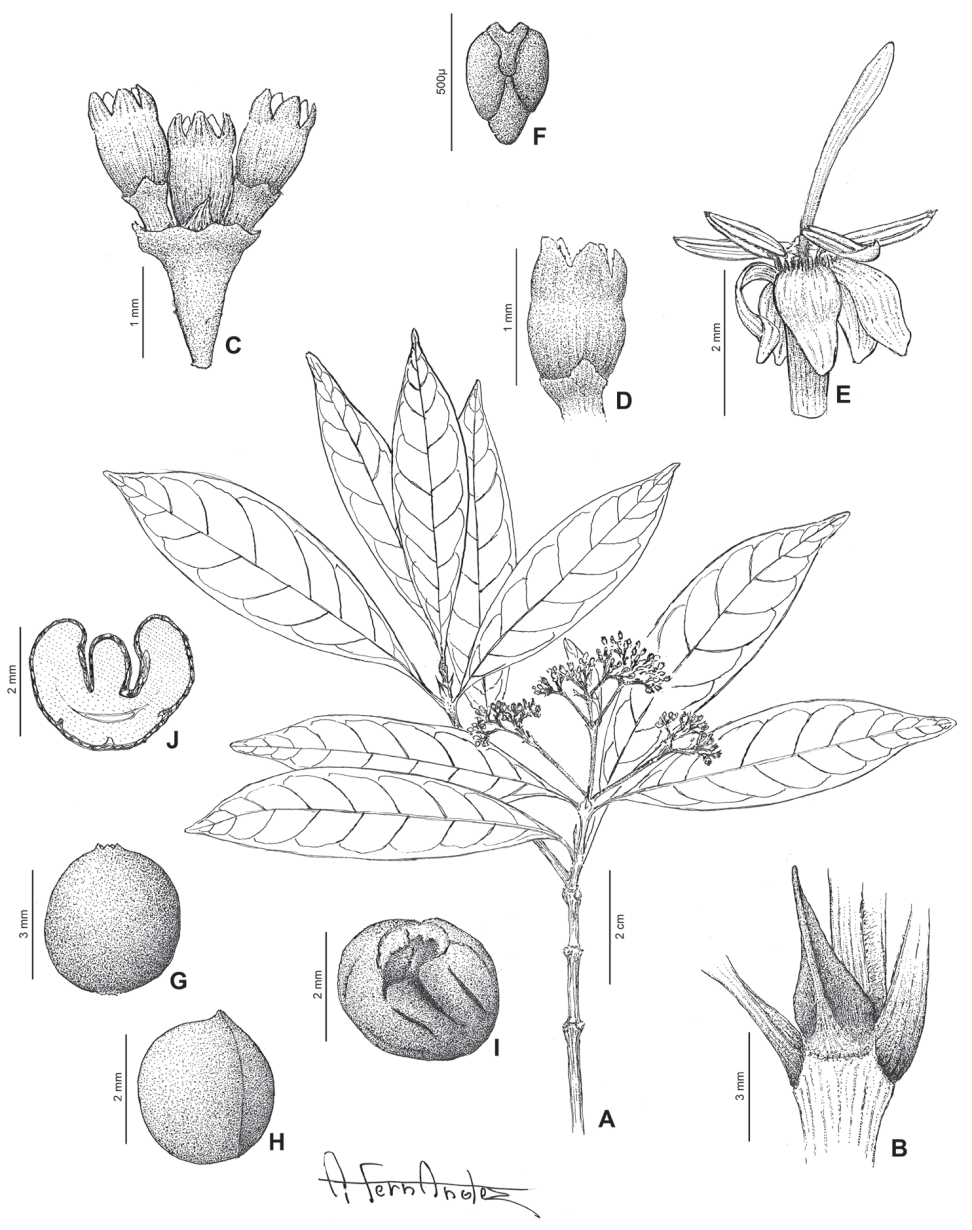
*Pseudocoptosperma menabense*. **A** fruiting branch **B** stipule **C** triad of flowers (corollas removed) **D** bracteole, ovary and calyx **E** corolla, style, stigma and anthers **F** placenta and ovules, abaxial view **G** fruit **H** pyrene **I** seed **J** longitudinal section through seed. **A, B** Groeninckx et al. 108 **C–F** Martin 8252-SF **G–J** Rabarivola 19861-SF.

Pyrenes are crustacous (*Pseudocoptosperma*, *Tulearia splendida*) or stony (*Exallosperma*, *Helictosperma* and *T. capsaintemariensis*). The pyrenes of *Pseudocoptosperma* (Fig. 10H) and *Tulearia* have a small central apical protuberance or ridge on the adaxial surface which is absent in pyrenes of *Exallosperma* and *Helictosperma*. In *Exallosperma*, the adaxial side of the pyrene is only a flat rim bordering a large opening (Fig. 5C, E). In all four genera, an opening mechanism for the pyrene exists. In *Pseudocoptosperma*, the single pyrene opens along the line of fusion of the locules (Fig. 10H). In *Tulearia*, the hemi-ovoidal pyrenes open along a central longitudinal preformed germination slit running over the entire length on the abaxial and adaxial sides (running through the apical ridge or the apical protuberance). In *Exallosperma*, the hemispherical pyrene opens along a short central longitudinal preformed germination slit situated apically on the abaxial and adaxial sides (Fig. 5B, C). The most intricate opening mechanism is found in *Helictosperma*. Here, the pyrenes open along four preformed longitudinal germination slits, two of which run along the lines of fusion of the locules and two of which are perpendicular to those. The pyrenes fall apart into four valves (Fig. 8A, B). A similar opening mechanism is found in two *Homollea* species (De Block 2018). We postulate that the opening mechanisms in the pyrenes are adaptations to growth in a dry environment, allowing seeds to be freed rapidly after a period of rain.

**Figure 11.**
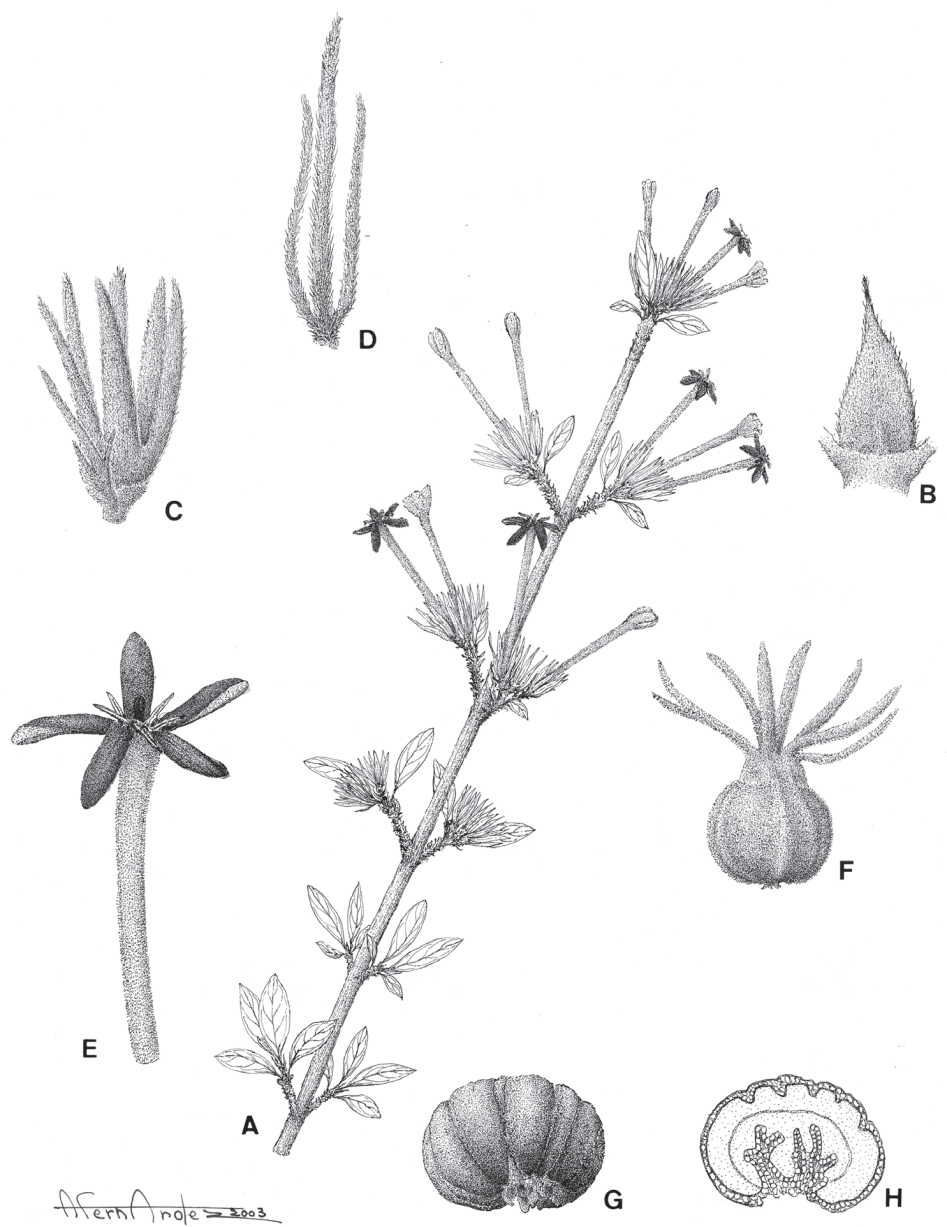
*Tulearia splendida*. A, flowering branch **B** stipule **C** bracteole, ovary and calyx **D** bract **E** corolla, style, stigma and anthers **F** fruit **G** seed **H** cross-section through seed. **A–E** De Block et al. 542 **F–H** Groeninckx et al. 209.

**Figure 12.**
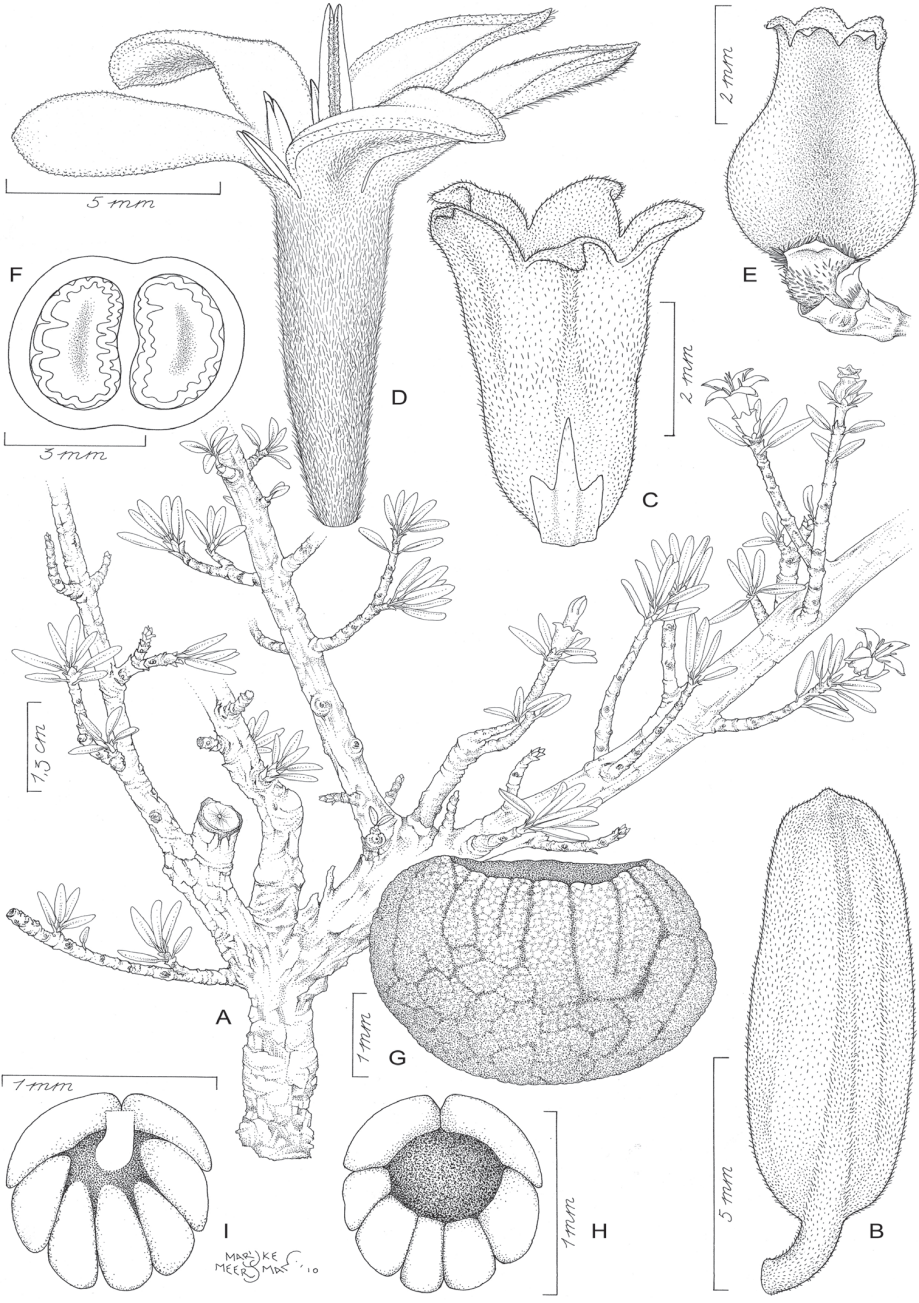
*Tulearia capsaintemariensis*. A, habit **B** adaxial view of leaf **C** bracteole, ovary and calyx **D** corolla, style, stigma and anthers **E** young fruit **F** transverse section through fruit **G** seed, lateral view **H** placenta and ovules, abaxial view **I** placenta and ovules, adaxial view. **A, B, E, F** Groeninckx et al. 309 **C, D, G–I** De Block et al. 2421.

**Seeds**. Seed size and shape is very variable within the Pavetteae, ranging from angular in, for example, *Leptactina* (with up to 100 seeds per fruit), to laterally flattened in *Homollea* and *Paracephaelis*, to hemispherical or hemiovoidal in, for example, *Pavetta* (with two seeds per fruit) and to (sub)spherical or ovoidal in, for example, *Coptosperma* (with a single seed per fruit). This variation is also present in the four new genera. *Pseudocoptosperma menabense* has a spherical seed (Fig. 10I), *Exallosperma* has laterally flattened seeds (Fig. 5D, E); *Tulearia* has hemispherical-hemiovoidal seeds (Figs 11G, H, 12G) and *Helictosperma* has seeds that in outline are spherical but are in fact rolled-in on themselves (Fig. 8C, D).

Two of the four new genera, *Pseudocoptosperma* and *Tulearia*, have ruminate seeds, a character that occurs in several other Pavetteae genera, notably in the Afro-Madagascan *Coptosperma*, the Madagascan *Robbrechtia* and *Schizenterospermum*, the continental African *Rutidea* (De Block 1995) and *Nichallea* and certain species of *Tarenna* in Asia. For the Afro-Madagascan genera of the Pavetteae, rumination hitherto was correlated with single-seeded fruits but, in *Tulearia*, two ruminate seeds are present per fruit. In Madagascar, having ruminate seeds is often correlated with growth in dry vegetation types (exception: *Robbrechtia*; De Block 2003). The surface enlargement of the seed-coat caused by rumination has been suggested to be beneficial for the intake of water by the seed (Boesewinkel and Bouman 1984).

**Seed-coat**. The seed-coat consists of an exotesta and an endotesta. As in most Pavetteae and Rubiaceae, the endotesta consists of several layers of thin-walled cells. In mature seeds, the cell layers of the endotesta are crushed into an amorphous layer by the growth of the endosperm. Sometimes, parts of the endotesta remain uncrushed in folds and undulations of the seed-coat in ruminate seeds. There is no difference in the endotesta of the four genera except for the presence or absence of crystals, which are abundant in *Helictosperma* and *Exallosperma* but absent in *Pseudocoptosperma* and *Tulearia*.

In the Pavetteae, the exotesta consists of a single cell layer and this is also the case in the new genera. The cells of the exotesta may be parenchymatous or thickened. The cell lumina are filled with tannins (Robbrecht and Puff 1986). In *Exallosperma* and *Helictosperma*, the exotesta cells have continuous plate-like thickenings along the outer tangential and the upper parts of the radial walls. In *Helictosperma*, in the region of the hilum, the thickened parts of the radial walls of the exotesta cells elongate, which results in a weakly thickened annulus around the hilum. This seed-coat type is also found in *Homollea* and *Paracephaelis* [Bridson and Robbrecht 1985: fig. 8, *Paracephaelis trichantha* (Baker) De Block as *Tarenna trichantha* (Baker) Bremek.]. In *Exallosperma*, the exotesta cells do not only elongate in the region of the hilum, but also in irregular lines, resulting in ridges across the seed surface (Fig. 5D, E). This situation is unique within the Pavetteae.

In *Pseudocoptosperma* and *Tulearia*, the exotesta cells are parenchymatous and filled with tannins. There is no elongation of the exotesta cells in the region of the hilum and an annulus is absent. Parenchymatous exotesta cells filled with tannins are also found in the other Pavetteae genera with ruminate seeds (De Block 1995; De Block 2003; De Block et al. 2001). It could be hypothesised that the tannins in the cell lumina of the exotesta cells, abundantly present because of the enlarged surface caused by rumination, have taken over the mechanical protection of the seed, eliminating the need for thickening of the exotesta cell wall. This could be explained by the fact that tannins contain phenolic substances that render the seed unpalatable for predators and protect it against pathogens (Tsou and Mori 2002).

**Figure 13.**
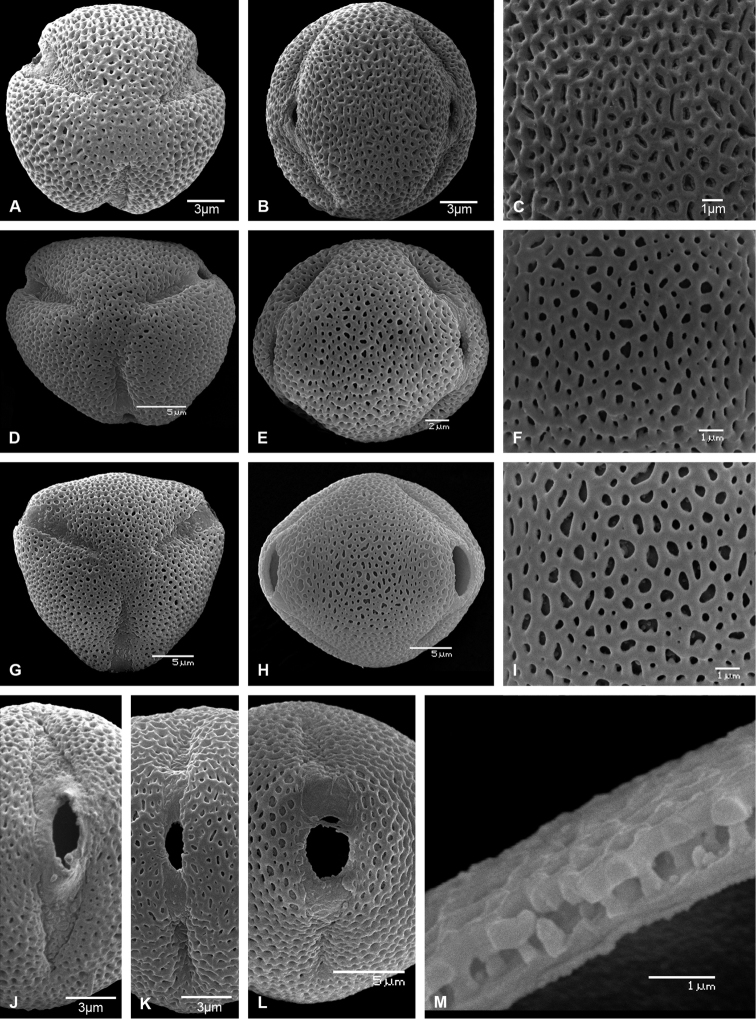
Pollen of *Pseudocoptosperma* and *Tulearia*. **A–C, J** *Pseudocoptosperma menabense* **D–F, K** *Tulearia capsaintemariensis* **G–I, L, M** *T. splendida*. **A, D, G** polar view **B, E, H** equatorial view **C, F, I** mesocolpium **J–L** ectoaperture **M** pollen grain wall. **A–C, J** Capuron 20569-SF; **D–F, K** Groeninckx et al. 309 **G–I, L, M** Capuron 20777-SF.

**Pollen**. *Exallosperma* (Fig. 9A–D, M), *Helictosperma* (Fig. 9E–L, N), *Pseudocoptosperma* (Fig. 13 A–C, J) and *Tulearia* (Fig. 13D–I, K–M) have pollen without supratectal elements. This is the common condition within the tribe Pavetteae. Supratectal elements are found in the two other subclades of clade VII, notably in *Homollea* and in *Coptosperma nigrescens* (but not in the other *Coptosperma* species in this clade). Supratectal elements are also found in the Afro-Madagascan genus *Paracephaelis* and in *Pavetta*, which is absent from Madagascar (De Block and Robbrecht 1998). The microreticulate to perforate tectum, which is present in *Helictosperma*, *Pseudocoptosperma* and *Tulearia*, is common within the tribe. *Exallosperma longiflora* is the only Pavetteae species with psilate tectum.

**Figure 14.**
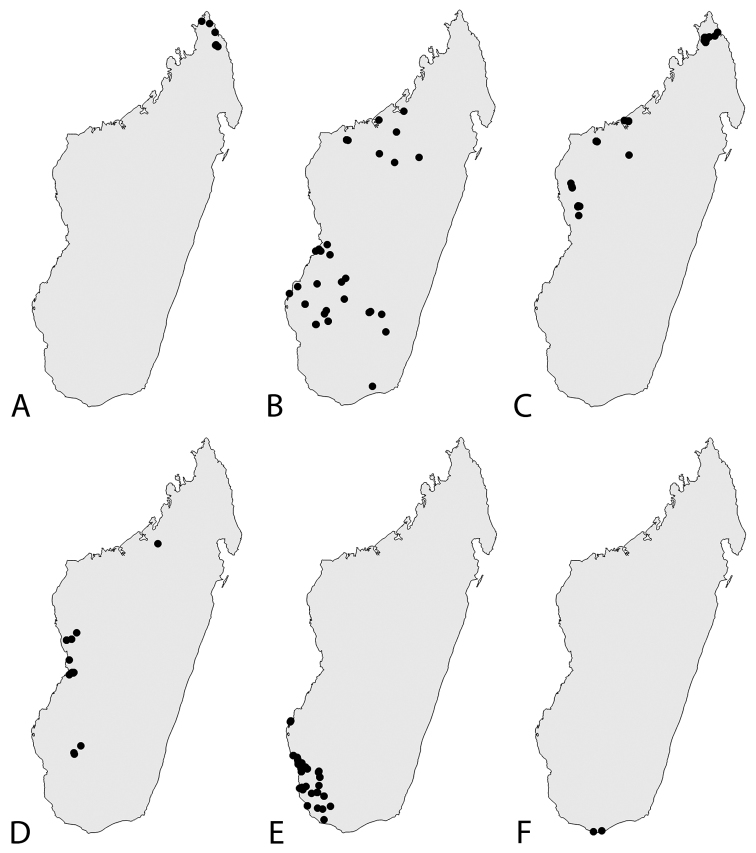
Distribution maps. **A** *Exallosperma longiflora* **B** *Helictosperma malacophylla* **C** *H. poissoniana* **D** *Pseudocoptosperma menabense* **E** *Tulearia splendida* **F** *T. capsaintemariensis*.

## Supplementary Material

XML Treatment for Exallosperma

XML Treatment for Exallosperma longiflora

XML Treatment for Helictosperma

XML Treatment for Helictosperma malacophylla

XML Treatment for Helictosperma poissoniana

XML Treatment for Pseudocoptosperma

XML Treatment for Pseudocoptosperma menabense

XML Treatment for Tulearia

XML Treatment for Tulearia splendida

XML Treatment for Tulearia capsaintemariensis

## Appendix 1

List of taxa used in the phylogenetic analyses with voucher information (geographic origin, collection, herbarium) and EMBL accession numbers for the plastid and nuclear markers *rps16*, *trnT-F*, ITS, *petD, accD*-*psa1* and *PI*. Previously published sequences are all from De Block et al. (2015) except for those indicated with ^(1)^ from Bremer and Eriksson (2009). New sequences are marked with *.

**Tribe Pavetteae** A.Rich. ex Dumort.: ***Coptosperma*** Hook.f.: ***C. borbonicum*** (Hend. & Andr.Hend.) De Block, Comores, De Block 1389 (BR), KM592189, KM592096, KM592283, MH175359*, MH175297*, MH175411*; ***C. graveolens*** (S.Moore) Degreef, Kenya, Mwachala 3711 (BR), KM592200, KM592107, KM592293, MH175360*, MH175298*, MH175412*; ***C. littorale*** (Hiern) Degreef, Mozambique, Luke & al. 9954 (UPS), KM592190, KM592097, KM592284, MH175361*, MH175299*, MH175413*; ***C. madagascariense*** (Baill.) De Block, Madagascar, Razafimandimbison & al. 577 (UPS), KM592191, KM592098, –, MH175362*, MH175300*, –; ***C. madagascariense*** (Baill.) De Block, Madagascar, De Block & al. 2238 (BR), –, –, KM592285, –, –, –; ***C. nigrescens*** Hook.f., Madagascar, De Block & al. 535 (BR), KM592192, KM592099, KM592286, MH175363*, MH175301*, MH175414*; ***C. nigrescens*** Hook.f., Kenya, Luke & Luke 9030 (UPS), KM592193, KM592100, KM592287, MH175364*, MH175302*, –; ***C. peteri*** (Bridson) Degreef, Tanzania, Lovett & Congdon 2991 (BR), KM592201, KM592108, KM592294, MH175365*, MH175303*, MH175415*; ***C. supra-axillare*** (Hemsl.) Degreef, Madagascar, De Block & al. 1321 (BR), KM592194, KM592101, KM592288, MH175366*, MH175304*, MH175416*; ***C.* sp. nov. B**, Madagascar, De Block & al. 796 (BR), KM592195, KM592102, KM592289, MH175367*, MH175305*, MH175417*; ***C.* sp. nov. C**, Madagascar, De Block & al. 1355 (BR), KM592196, KM592103, KM592290, MH175368*, MH175306*, MH175418*; ***C.* sp. nov. D**, Madagascar, De Block & al. 704 (BR), KM592197, KM592104, KM592291, MH175369*, MH175307*, MH175419*; ***C.* sp. nov. E**, Madagascar, De Block & al. 733 (BR), KM592198, KM592105, –, MH175370*, MH175308*, MH175420*. – ***Exallosperma*** De Block: ***E. longiflora*** De Block, Madagascar, De Block et al. 1080 (BR), MH175452*, MH175464*, MH175348*, MH175371*, MH175309*, –; Madagascar, Nusbaumer & Ranirison 1992 (G), MH175453*, MH175465*, MH175349*, MH175372*, MH175310*, –. – ***Helictosperma*** De Block: ***H. malacophylla*** (Drake) De Block, Madagascar, De Block et al. 534 (BR), MH175454*, MH175466*, MH175350*, MH175373*, MH175311*, –; Madagascar, De Block et al. 2194 (BR), MH175455*, MH175467*, MH175351*, MH175374*, MH175312*, MH175421*; ***H. poissoniana*** Homolle ex De Block, Madagascar, De Block et al. 797 (BR), MH175456*, MH175468*, MH175352*, MH175375*, MH175313*, MH175422*. – ***Homollea*** Arènes: ***H. leandrii*** Arènes, Madagascar, Andriambololonera & al. 171 (BR), –, –, MH175353*, MH175376*, –, –; ***H. longiflora*** Arènes, Madagascar, De Block & al. 767 (BR), KM592205, KM592112, KM592296, MH175377*, MH175314*, MH175423*; ***H. perrieri*** Arènes, Madagascar, Morat 4700 (TAN), KM592206, KM592113, KM592297, MH175377*, MH175315*, MH175424*. – ***Leptactina*** Hook.f.: ***L. mannii*** Hook.f., Gabon, Dessein & al. 2518 (BR), KM592214, KM592121, KM592302, MH175379*, MH175316*, MH175425*. – ***Paracephaelis*** Baill.: ***P. cinerea*** (A.Rich. ex DC.) De Block, Madagascar, De Block & al. 2193 (BR), KM592220, KM592127, KM592308, MH175380*, MH175317*, MH175426*; ***P. saxatilis*** (Scott-Elliot) De Block, Madagascar, Davis & al. 2731 (K), KM592221, KM592128, –, MH175381*, MH175318*, –; ***P. saxatilis*** (Scott-Elliot) De Block, Madagascar, De Block & al. 2401 (BR), –, – KM592309, –, –, –; ***P. sericea*** (Arènes) De Block, Madagascar, De Block & al. 849 (BR), KM592207, KM592114, KM592298, MH175382*, MH175319*, –; ***P. tiliacea*** Baill., Madagascar, Groeninckx & al. 113 (BR), KM592222, KM592129, KM592310, MH175383*, MH175320*, MH175427*. – ***Pseudocoptosperma*** De Block: ***P. menabense*** Capuron ex De Block: Madagascar, Davis et al. 2564 (K), MH175457*, MH175469*, MH175354*, MH175384*, MH175321*, MH175428*; Madagascar, Razafimandimbison & Bremer 487 (UPS), MH175458*, MH175470*, MH175355*, MH175385*, MH175322*, MH175429*. – ***Robbrechtia*** De Block: ***R. grandifolia*** De Block, Madagascar, Kårehed 311 (UPS), AM117339^(1)^, AM117383^(1)^, KM592325, MH175386*, MH175323*, MH175430*; ***R. milleri*** De Block, Madagascar, Bremer & al. 5295 (S), KM592240, KM592147, KM592326, MH175387*, MH175324*, MH175431*. – ***Schizenterospermum*** Homolle ex Arènes: ***S. grevei*** Homolle ex Arènes, Madagascar, De Block & al. 2167 (BR), KM592250, KM592156, KM592333, MH175388*, MH175325*, MH175432*; ***S. rotundifolia*** Homolle ex Arènes, Madagascar, De Block & al. 771 (BR), KM592251, KM592157, KM592334, MH175389*, MH175326*, MH175433*. – ***Tarenna*** Gaertn.: ***T. alleizettei*** (Dubard & Dop) De Block, Madagascar, De Block & al. 1883 (BR), KM592272, KM592178, KM592353, MH175390*, MH175327*, MH175434*; ***T. alpestris*** (Wight) N.P.Balakr., India, De Block 1474 (BR), KM592252, KM592158, KM592335, MH175391*, MH175328*, MH175435*; ***T. asiatica*** (L.) Kuntze ex K.Schum., India, Auroville 998 (SBT), KM592253, KM592159, KM592336, MH175392*, MH175329*, MH175436*; ***T. attenuata*** (Hook.f.) Hutch., Asia, country unknown, BR Living Collection 20031135-53 (BR), KM592254, KM592160, KM592337, –, –, –; ***T. capuroniana*** De Block, Madagascar, De Block & al. 937 (BR), KM592273, KM592179, KM592354, MH175393*, MH175330*, MH175437*; ***T. depauperata*** Hutch., China, Chow & Wan 79063 (UPS), KM592256, KM592162, KM592339, MH175394*, MH175331*, MH175438*; ***T. flava*** Alston, Sri Lanka, Klackenberg 440 (S), KM592257, KM592163, KM592340, MH175395*, MH175332*, MH175439*; ***T. gracilipes*** (Hayata) Ohwi, Japan, Van Caekenberghe 149 (BR), KM592259, KM592165, –, MH175396*, MH175333*, –; ***T. grevei*** (Drake) Homolle, Madagascar, De Block & al. 959 (BR), KM592274, KM592180, KM592355, MH175397*, MH175334*, –; ***T. leioloba*** (Guillaumin) S.Moore, New Caledonia, Mouly 174 (P), KM592262, KM592168, KM592343, MH175398*, MH175335*, MH175440*; ***T. microcarpa*** (Guillaumin) Jérémie, New Caledonia, Mouly 297 (P), KM592263, KM592169, KM592344, MH175399*, MH175336*, MH175441*; ***T. precidantenna*** N.Hallé, Gabon, Dessein & al. 2360 (BR), KM592267, KM592173, KM592348, MH175400*, MH175337*, MH175442*; ***T. rhypalostigma*** (Schltr.) Bremek., New Caledonia, Mouly 182 (P), KM592268, KM592174, KM592349, MH175401*, MH175338*, MH175443*; ***T. sambucina*** (G.Forst.) T.Durand ex Drake, New Caledonia, Mouly & al. 364 (P), KM592270, KM592176, KM592351, MH175402*, MH175339*, MH175444*; ***T. spiranthera*** (Drake) Homolle, Madagascar, De Block & al. 946 (BR), KM592275, KM592181, KM592356, MH175403*, MH175340*, –; ***T. thouarsiana*** (Drake) Homolle, Madagascar, De Block & al. 655 (BR), KM592276, KM592182, KM592357, MH175404*, MH175341*, MH175445*; ***T. uniflora*** (Drake) Homolle, Madagascar, Bremer & al. 5230 (S), KM592277, KM592183, KM592358, MH175405*, MH175342*, MH175446*. – ***Tennantia*** Verdc.: ***T. sennii*** (Chiov.) Verdc. & Bridson, Kenya, Luke & al. 8357 (UPS), KM592278, KM592184, KM592359, MH175406*, MH175343*, MH175447*. – ***Tulearia*** De Block: ***T. capsaintemariensis*** De Block, Madagascar, De Block et al. 2421 (BR), MH175459*, MH175471*, MH175356*, MH175407*, MH175344*, MH175448*; Madagascar, Groeninckx et al. 309 (BR), MH175460*, MH175471*, MH175357*, MH175408*, MH175345*, MH175449*; ***T. splendida*** De Block, Madagascar, De Block et al. 542 (BR), MH175461*, MH175473*, –, MH175409*, MH175346*, MH175450*; Madagascar, De Block et al. 2287 (BR), MH175462*, MH175474*, MH175358*, MH175410*, MH175347*, MH175451*; Madagascar, Razafimandimbison 526 (UPS), MH175463*, MH175475*, –, –, –, –.
